# Clinical determinants of early parasitological response to ACTs in African patients with uncomplicated falciparum malaria: a literature review and meta-analysis of individual patient data

**DOI:** 10.1186/s12916-015-0445-x

**Published:** 2015-09-07

**Authors:** WWARN Artemisinin based Combination Therapy (ACT) Africa Baseline Study Group

**Affiliations:** Nuffield Department of Clinical Medicine, WorldWide Antimalarial Resistance Network (WWARN), Centre for Tropical Medicine and Global Health, University of Oxford, Oxford, UK

## Abstract

**Background:**

Artemisinin-resistant *Plasmodium falciparum* has emerged in the Greater Mekong sub-region and poses a major global public health threat. Slow parasite clearance is a key clinical manifestation of reduced susceptibility to artemisinin. This study was designed to establish the baseline values for clearance in patients from Sub-Saharan African countries with uncomplicated malaria treated with artemisinin-based combination therapies (ACTs).

**Methods:**

A literature review in PubMed was conducted in March 2013 to identify all prospective clinical trials (uncontrolled trials, controlled trials and randomized controlled trials), including ACTs conducted in Sub-Saharan Africa, between 1960 and 2012. Individual patient data from these studies were shared with the WorldWide Antimalarial Resistance Network (WWARN) and pooled using an *a priori* statistical analytical plan. Factors affecting early parasitological response were investigated using logistic regression with study sites fitted as a random effect. The risk of bias in included studies was evaluated based on study design, methodology and missing data.

**Results:**

In total, 29,493 patients from 84 clinical trials were included in the analysis, treated with artemether-lumefantrine (n = 13,664), artesunate-amodiaquine (n = 11,337) and dihydroartemisinin-piperaquine (n = 4,492). The overall parasite clearance rate was rapid. The parasite positivity rate (PPR) decreased from 59.7 % (95 % CI: 54.5–64.9) on day 1 to 6.7 % (95 % CI: 4.8–8.7) on day 2 and 0.9 % (95 % CI: 0.5–1.2) on day 3. The 95th percentile of observed day 3 PPR was 5.3 %. Independent risk factors predictive of day 3 positivity were: high baseline parasitaemia (adjusted odds ratio (AOR) = 1.16 (95 % CI: 1.08–1.25); per 2-fold increase in parasite density, *P* <0.001); fever (>37.5 °C) (AOR = 1.50 (95 % CI: 1.06–2.13), *P* = 0.022); severe anaemia (AOR = 2.04 (95 % CI: 1.21–3.44), *P* = 0.008); areas of low/moderate transmission setting (AOR = 2.71 (95 % CI: 1.38–5.36), *P* = 0.004); and treatment with the loose formulation of artesunate-amodiaquine (AOR = 2.27 (95 % CI: 1.14–4.51), *P* = 0.020, compared to dihydroartemisinin-piperaquine).

**Conclusions:**

The three ACTs assessed in this analysis continue to achieve rapid early parasitological clearance across the sites assessed in Sub-Saharan Africa. A threshold of 5 % day 3 parasite positivity from a minimum sample size of 50 patients provides a more sensitive benchmark in Sub-Saharan Africa compared to the current recommended threshold of 10 % to trigger further investigation of artemisinin susceptibility.

**Electronic supplementary material:**

The online version of this article (doi:10.1186/s12916-015-0445-x) contains supplementary material, which is available to authorized users.

## Background

The increasing availability of artemisinin-based combination therapies (ACTs) and long-lasting insecticidal nets (LLINs) over the last decade has contributed to a substantial reduction in malaria morbidity and mortality in Sub-Saharan Africa (SSA) [1, 2]. However, the reduced efficacy of artemisinin against *Plasmodium falciparum* malaria in the Greater Mekong region [3–9] threatens to jeopardize the recent gains in malaria control and elimination. Identifying areas where decreased artemisinin susceptibility is emerging is critical to inform an adequate international response.

Delayed parasite clearance is the hallmark of artemisinin resistance [4, 10, 11]. However, its precise measurement requires frequent sampling and this is often logistically difficult to implement in resource-constrained settings [12]. Recently, specific mutations in the Kelch 13 (K13) gene have been shown to be highly correlated with the slow clearance phenotype in parasites from Northwest Cambodia [13] and other parts of the Greater Mekong sub-region [8, 14]. Although K13 mutations are present in Africa, the variants differ from those in Southeast Asia and their correlation with artemisinin resistance has yet to be substantiated [15–18]. The proportion of patients with persistent patent parasitaemia (parasite positivity rate, PPR) on day 3 has been proposed as a simple and pragmatic metric of choice for routine monitoring to identify suspected artemisinin resistance [19]. In depth clinical and parasitological assessments are warranted in sites where parasite positivity rate on day 3 (72 hours) exceeds 10 % in a study [19]. If less than 3 % of the patients in a site are still parasitaemic on day 3, artemisinin resistance is considered highly unlikely [20]. This threshold has been developed with data mostly from low transmission settings in Southeast Asia [20].

It is known that the speed of parasite clearance is influenced by a number of host, parasite and drug factors [10, 11, 21], including the level of acquired immunity [22–24], parasite density at presentation [20, 25–27], the quality of microscopy [28], the pharmacokinetic/pharmacodynamic profiles of the different artemisinin derivatives and the partner drugs [29].

Therefore, to assess the dynamics of early parasitological response after artemisinin combination therapy observed in SSA, parasite clearance data were compiled from patients with uncomplicated *P. falciparum* malaria enrolled in ACT clinical efficacy trials conducted between 1999 and 2012. The aim was to provide a baseline of early parasitological response profiles so that sites at high risk (hot spots) for artemisinin resistance can be identified going forward, to inform malaria control and containment efforts.

## Methods

### Identification of studies for potential inclusion

#### Individual patient data

A literature review was conducted in PubMed in March 2013 and updated in 2014 to identify all published clinical trials of antimalarials since 1960. All antimalarial clinical trials published since 1960 were identified by the application of the key terms ((malaria OR plasmod*) AND (amodiaquine OR atovaquone OR artemisinin OR arteether OR artesunate OR artemether OR artemotil OR azithromycin OR artekin OR chloroquine OR chlorproguanil OR cycloguanil OR clindamycin OR coartem OR dapsone OR dihydroartemisinin OR duo-cotecxin OR doxycycline OR halofantrine OR lumefantrine OR lariam OR malarone OR mefloquine OR naphthoquine OR naphthoquinone OR piperaquine OR primaquine OR proguanil OR pyrimethamine OR pyronaridine OR quinidine OR quinine OR riamet OR sulphadoxine OR tetracycline OR tafenoquine)) through the PubMed library. All references containing any mention of antimalarial drugs were tabulated and manually checked to confirm prospective clinical trials. Studies on prevention or prophylaxis, reviews, animal studies or studies of patients with severe malaria or in pregnant women were excluded. When pdfs were available further details of the publications were reviewed, and basic details on the study methodology, treatment arms assessed and the study locations were documented. These are provided in the WorldWide Antimalarial Resistance Network (WWARN) publication library [30]. Specific details of the studies with ACTs are available in Additional files 1 and 2. The year of the study was taken as the year in which the paper was published, although the start and end date of patient enrolment were also recorded. Where a specific site was not reported in the manuscript, the capital city of the country was used as the default location. Countries were grouped into four sub-regions: East; West; Central; and South Africa, as reported in the WHO *World malaria report 2014* [1].

All research groups in the systematic review were contacted to share the entire dataset of their study with WWARN. Those who had contributed studies previously to the WWARN data repository were also invited to participate and asked whether they were aware of any unpublished or ongoing clinical trials involving ACTs, and these additional unpublished studies were also requested. Studies were included in the meta-analysis provided that they were: i) prospective clinical efficacy studies of uncomplicated *P. falciparum* (either alone or mixed infections with *P. vivax*); ii) clinical trials conducted in SSA with one of the following three ACTs: artemether-lumefantrine (AL) (six-dose), dihydroartemisinin-piperaquine (DP) and one of the three formulations of artesunate-amodiaquine (AS-AQ): fixed dose combination (ASAQ-FDC), non-fixed dose combination in a loose formulation (ASAQ-loose NFDC) or non-fixed dose combination in a co-blister formulation (ASAQ-coblistered NFDC); and iii) parasitaemia was sampled at least on days 2 (48 hours) and 3 (72 hours) following treatment. Individual study protocols were available for all trials included, either from the publication or as a metafile submitted with the raw data. All data were uploaded to the WWARN repository and standardized using a methodology described in the clinical module data management and statistical analysis plan [31].

### Definition of parameters assessed

#### Anaemia

Anaemia was defined according to WHO guidelines [32] (that is, haemoglobin cut-offs for moderate anaemia were 10 g/dl in children <5 years of age and 11 g/dl in older patients, and for severe anaemia were 7 and 8 g/dl, respectively). For studies where only haematocrit was measured, the following relationship was used to estimate haemoglobin: Haematocrit (%) = 5.62 + 2.60 × Haemoglobin (g/dl) [33].

#### Parasite positivity

A pre-defined algorithm was used to impute positivity status on days 2 or 3, if no observation of the blood film was recorded on that day [34]. For studies with frequent sampling, a patient was classified as being positive on days 1, 2 and 3 after enrolment if the measurements within a window of ± 3 hours of 24, 48 and 72 hours were positive.

#### Malaria transmission intensity

The study sites were classified into two categories, low/moderate and high malaria transmission, based on the observed re-infection rate and the parasite prevalence estimates obtained from the Malaria Atlas Project [35]. More information about this classification is available in Additional file 3.

### Ethical approval

All data included in this analysis were obtained in accordance with ethical approvals from the country of origin. Ethical approval for pooled analysis of individual participant data was granted by the Oxford Tropical Research Ethics Committee (OxTREC), based on the fact that all studies contributed to WWARN must have already obtained all necessary ethical approvals and informed consent.

### Statistical analysis

All statistical analyses were carried out based on an a priori statistical plan [34]. The primary endpoint used in the analysis was microscopically defined parasite positivity on days 1, 2 and 3. The proportions of patients remaining parasitaemic on days 1, 2 and 3 were expressed as parasite positivity rates (PPRs) and were calculated for each study site separately using the individual patient data. The overall PPRs were calculated as a weighted average of the estimates from each of the individual study sites and associated confidence intervals (95 % CI) calculated by adjusting for within study clustering using the method described by Fleiss et al. [36]. Assuming baseline day 3 PPR equal to the upper limit of the 95 % CI around the observed PPR, we computed the maximum number of positive cases needed to be observed for the estimated 95 % CI to exclude this baseline for a given sample size, as described elsewhere [20].

Univariable and multivariable analyses of risk factors associated with parasite positivity status on days 1, 2 and 3 were conducted using generalized linear mixed model (logit link), in a one-stage analysis by combining all of the individual patient data. In order to account for within study clustering, study sites were fitted as random effects; the statistical significance of which was assessed using a likelihood ratio test. Statistical heterogeneity was quantified as the variance of the random effects using maximum likelihood method and the proportion of total variance contributed by the site-level variance component (ρ) was reported. Missing covariates were dealt with using multiple imputation methods. The number of imputations (*m*) was determined based on the fraction of missing information (γ) assuming 5 % loss in efficiency (*η*) using *m* ≥ γ*(*η*/1–*η*) [37]. Known confounders (age, parasitaemia and transmission setting) were kept in the model regardless of significance. Covariates examined at baseline included age, gender, fever (axillary, tympanic or rectal temperature >37.5 °C), parasitaemia, anaemia, gametocytemia, transmission setting, ACTs used for treatment, geographical region and year of the study. Any variables significant in univariable analysis (below 10 % level of significance) were kept for multivariable analysis; the decision of inclusion in the final model was assessed using a likelihood ratio test. In a sub-group of studies in which information was available on drug dosing, the effects of weight-adjusted doses (mg/kg) on parasite positivity status were evaluated after adjusting for the covariates significant in the multivariable analysis.

The robustness of the coefficients in the final multivariable model was examined using bootstrap sampling. Sensitivity analysis was performed by excluding one study site at a time and the coefficient of variation around the parameter estimates was calculated. The final model was used to simulate outcome for each patient and the observed PPRs were plotted against the simulated PPRs to assess model adequacy.

Continuous variables were compared between groups using generalized linear regression with study sites fitted as random effects. Data that were not normally distributed were compared with Mann–Whitney *U* test or Kruskal–Wallis test. All statistical analyses were carried out using R (version 3.1.2, R Foundation for Statistical Computing, Vienna, Austria) using *lme4* package.

### Assessment of risk of potential bias

In accordance with the Preferred Reporting Items for Systematic Reviews and Meta-Analyses (PRISMA) guidelines, the risk of bias within studies was assessed based on: 1) study design (randomization, sequence generation, blinding); 2) microscopy methodologies for parasite quantification; and 3) the proportion of patients with (a) missing outcomes (missing outcome on days 2 and 3) and (b) missing baseline covariates (age, temperature, haemoglobin/haematocrit).

To assess whether the non-availability of some individual participant data could have biased the results, we extracted data on PPRs from studies not providing individual patient data and performed a two-stage meta-analysis of proportions using logit transformation; a continuity correction of 0.5 was applied to studies with zero cell count using *meta* package. Publication bias was assessed through the use of a funnel plot of the log-transformed odds ratio, the asymmetry of which was tested using Egger’s method.

## Results

### Characteristics of eligible studies

**Fig. 1:**
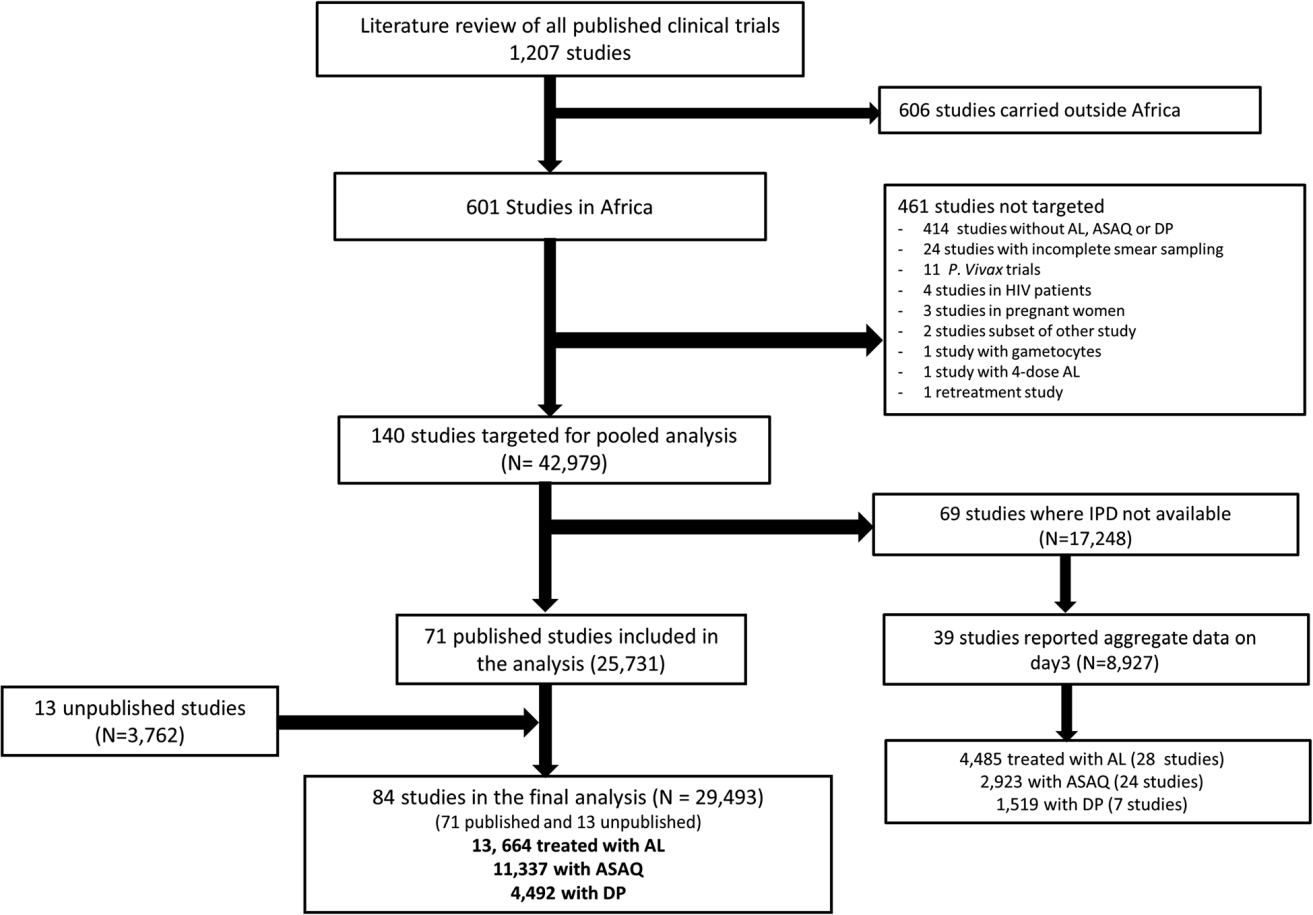
Patient flowchart. AL, artemether-lumefantrine; AS-AQ, artesunate-amodiaquine; DP, dihydroartemisinin-piperaquine; IPD, individual participant data

The systematic literature review identified 140 published clinical studies of ACT efficacy that were potentially relevant to this analysis. Researchers agreed to share individual patient data from 71 trials (50.7 %) including 25,731 patients (59.9 % of the targeted population). Additional data were available for 3,762 patients from 13 unpublished trials. In total, individual records were available from 29,493 patients enrolled in 27 different countries between 1999 and 2012 (Fig. 1). Fourteen studies (n = 4,177) had a single arm and the remaining 70 studies had at least two ACT arms (n = 25,376). Among these, 65 studies were randomized, 14 were non-randomized and randomization status was not reported in 5 studies. AL was administered to 46 % (n = 13,664) and DP to 15 % (n = 4,492) of patients. AS-AQ was administered in three different formulations: ASAQ-FDC (17 %, n = 4,907); ASAQ-loose NFDC (13 %, n = 3,925); and ASAQ-coblistered NFDC (9 %, n = 2,505). Thirty-five studies were conducted in West Africa (n = 10,676), 31 in East Africa (n = 8,331), 4 in Central Africa (n = 609), 4 in South Africa (n = 666), and the remaining 10 studies were multi-regional (n = 9,211).

### Baseline characteristics

The baseline characteristics of the included patients are given in Table 1. The mean age (years ± SD) was 6.7 ± 8.78, and was similar for patients treated with AL (7.4 ± 9.22) and AS-AQ (6.6 ± 8.60). The mean age was lower for patients treated with DP (4.9 ± 7.51), with 90 % (4,064/4,492) of patients treated with this regimen being less than 12 years old (*P* <0.05, linear regression). The median baseline parasitaemia was 20,200 parasites/μl (IQR: 6,320–51,520) with slight differences between treatment groups (Table 1). A high proportion (55.5 %, 11,918/21,479) of patients were anaemic at enrolment and 9 % (2,083/22,402) of the patients carried gametocytes at presentation (Table 1). After adjustment for age, both of these percentages were similar in the different treatment groups.

**Table 1:** Baseline characteristics of the patients in the analysis

Baseline characteristics	AL (2002–2012)	AS-AQ (1999–2012)	DP (2003–2011)	Total (1999–2012)
Patients (N)	13,664 (46.3 %)	11,337 (38.4 %)	4,492 (15.2 %)	29,493
Female	6,437 (47.1 %)	5,322 (46.9 %)	2,123 (47.3 %)	13,882 (47.1 %)
Age				
Mean age ± SD (years)	7.4 ± 9.22	6.6 ± 8.60	4.9 ± 7.51	6.7 ± 8.78
<1 year	795 (5.8 %)	842 (7.4 %)	447 (10.0 %)	2,084 (7.1 %)
1 to <5 years	7,183 (52.6 %)	6,324 (55.8 %)	3,185 (70.9 %)	16,692 (56.6 %)
5 to <12 years	3,184 (23.3 %)	2,357 (20.8 %)	432 (9.6 %)	5,973 (20.3 %)
≥12 years	2,478 (18.1 %)	1,801 (15.9 %)	427 (9.5 %)	4,706 (16.0 %)
Geographic region				
East Africa	6,040 (44.2 %)	2,920 (25.8 %)	2,229 (49.6 %)	11,189 (37.9 %)
West Africa	6,481 (47.4 %)	6,749 (59.5 %)	1,302 (29.0 %)	14,532 (49.3 %)
Central Africa	483 (3.5 %)	758 (6.7 %)	174 (3.9 %)	1,415 (4.8 %)
South Africa	660 (4.8 %)	910 (8.0 %)	787 (17.5 %)	2,357 (8.0 %)
Transmission settings				
High	4,836 (35.4 %)	4,062 (35.8 %)	1,876 (41.8 %)	10,774 (36.5 %)
Low/moderate	8,828 (64.6 %)	7,275 (64.2 %)	2,616 (58.2 %)	18,719 (63.5 %)
Enrolment clinical parameters				
Mean body weight ± SD (kg)	21.2 ± 16.23	19.5 ± 15.26	16.3 ± 13.72	19.8 ± 15.59
Median parasitaemia (IQR)	19,260 (5,930–48,260)	20,000 (6,080–52,480)	25,540 (8,320–59,830)	20,200 (6,320–51,520)
Parasitaemia >100,000/μL	8.4 % (1,152/13,664)	10.7 % (1,209/11,337)	11.7 % (527/4,492)	9.8 % (2,888/29,493)
Mean haemoglobin ± SD (g/dl)	10.3 ± 2.17	9.7 ± 2.10	9.6 ± 1.86	9.9 ± 2.11
Gametocytes presence	8.2 % (868/10,649)	11.1 % (821/7,428)	9.1 % (394/4,325)	9.3 % (2,083/22,402)
Elevated temperature (>37.5 °C)	61.9 % (7,861/12,691)	67.3 % (7,461/11,092)	63.7 % (2,814/4,419)	64.3 % (18,136/28,202)
Anaemia				
Moderate	44 % (4,246/9,650)	48.6 % (3,761/7,734)	52.7 % (2,159/4,095)	47.3 % (10,166/21,479)
Severe	6.9 % (666/9,650)	10.1 % (780/7,734)	7.5 % (306/4,095)	8.2 % (1,752/21,479)

AL, artemether-lumefantrine; AS-AQ, artesunate-amodiaquine; DP, dihydroartemisinin-piperaquine

### Observed parasite positivity rates (PPRs) on days 1, 2 and 3

**Fig. 2:**
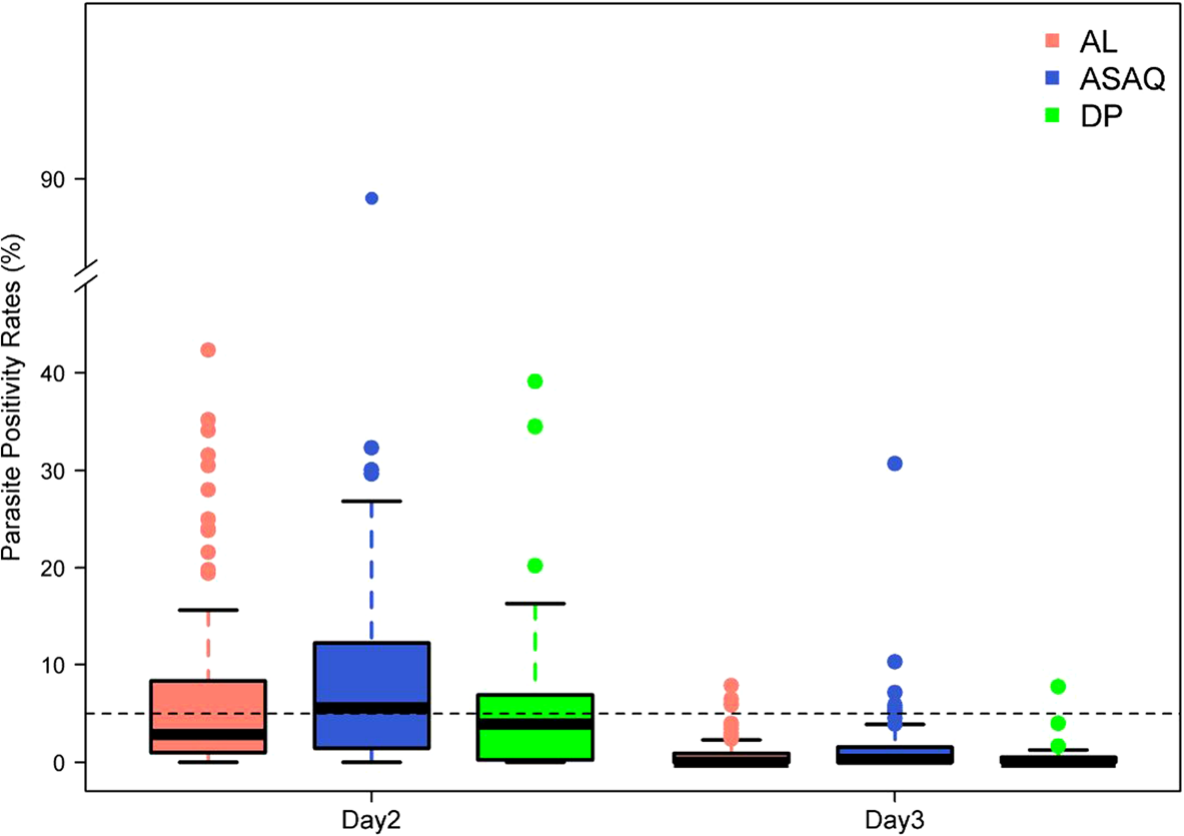
Parasite positivity rates (PPRs) on days 2 and 3 following treatment administration. Boxplot showing PPRs for each of the ACTs separately. Only studies with sample size >25 patients were considered for the plot. There were two study sites with day 3 PPR >10 %, both of these sites used the non-fixed presentations of AS-AQ. ACT, artemisinin-based combination therapy; AL, artemether-lumefantrine; AS-AQ, artesunate-amodiaquine; DP, dihydroartemisinin-piperaquine; PPR, parasite positivity rate

The presence and density of parasites on day 1 could only be assessed in 55 % (16,196/29,493) of patients (52 studies). The overall parasite clearance rate for all studies was rapid. The PPR decreased from 59.7 % (95 % CI: 54.5–64.9) on day 1 (10,099/16,916) to 6.7 % (95 % CI: 4.8–8.7) on day 2 (1,853/27,496) and 0.9 % (95 % CI: 0.5–1.2) on day 3 (253/28,580). The PPRs on days 1, 2 and 3 were similar for AL, DP and ASAQ-FDC, but higher for the non-fixed formulations of AS-AQ on days 2 and 3 (Table 2). Compared to patients older than 12 years, children from 1 to 5 years had the highest PPR on day 1 (64 %, 6,430/10,053, *P* <0.001) and day 2 (7.5 %, 1,176/15,677, *P* <0.001), but there was no age-related difference on day 3. Patients with an initial parasite density >100,000 parasites/μl had a PPR of 82.7 % (1,494/1,807) on day 1, 14.3 % (385/2,696) on day 2 and 1.3 % (37/2,752) on day 3. The corresponding proportions for patients with parasitaemia less than 100,000 parasites/μl were 57.0 % (8,605/15,109), 5.9 % (1,468/24,800) and 0.8 % (216/25,828), respectively for days 1, 2 and 3 (all *P* <0.05). There were no regional differences or temporal trend in the PPRs on any days during the time period studied, that is, 1999–2012. A detailed summary of the PPRs for each of the treatment regimens stratified by country and calendar year is presented in Additional file 4. In total, there were 22 sites that had a PPR on day 3 exceeding 3 % (Table 3). The risk of day 3 parasitaemia exceeding 3 % was greatest in patients treated with ASAQ-loose NFDC (19.0 %, 8/42) and ASAQ-coblistered NFDC (11.1 %, 1/9) compared to 9.4 % (3/32) for AS-AQ FDC, 5.6 % (2/36) for DP and 7.6 % (8/105) for AL (Table 3). At two sites, the day 3 PPR was higher than 10 %: Miandrivazo, Madagascar, 2006 (n = 68, PPR = 10.3 %, ASAQ-loose NFDC) and Yaoundé, Cameroon, 2005 (n = 101, PPR = 30.1 %, ASAQ-coblistered NFDC) (Fig. 2).

**Table 2:** Parasite positivity rate (PPR) for three different ACTs

	AL	AS-AQ^c^	DP	Overall
Day 1				
PPR (%)^a^	59.3 % (4,721/7,966) (95 % CI: 52.2–66.3)	60.3 % (3,463/5,746) (95 % CI: 54.7–65.8)	59.8 % (1,915/3,204) (95 % CI: 50.3–69.2)	59.7 % (10,099/16,916) (95 % CI: 54.5–64.9)
Number of study sites^b^	81	52	25	158
Median PPR (IQR; range)^b^	61.8 % (35.5–79.1; 0–97.6)	58.8 % (47.1–77.0; 0.0–96.3)	53.8 % (32.4–69.4; 18.3–93.0)	57.9 % (36.1–77.0; 0.0–97.6)
Day 2				
PPR (%)^a^	5.9 % (729/12,255)	7.2 % (784/10,821)	7.7 % (340/4,420)	6.7 % (1,853/27,496)
Number of study sites^b^	100	79	36	215
Median PPR (IQR; range)^b^	2.9 (1–8.3; 0.0–42.4)	5.6 % (1.5–12.3; 0.0–88.1)	3.9 % (0.4–6.7; 0.0–39.1)	3.3 % (1.2–10.2; 0.0–88.1)
Day 3				
PPR (%)^a^	0.6 % (76/13,004)	1.3 % (143/11,142)	0.8 % (34/4,434)	0.9 % (253/28,580)
Number of study sites^b^	105	84	36	225
Median PPR (IQR; range)^b^	0.0 % (0.0–0.9; 0.0–7.8)	0.3 % (0.0–1.6; 0.0–30.7)	0.0 % (0.0–0.5; 0.0–7.7)	0.0 % (0.0–0.7;0.0–30.7)

^a^The PPR was computed using all available data and associated 95 % confidence interval was adjusted for within site correlation; ^b^only sites with the number of patients >25 were considered; ^c^PPRs (95 % CI) on days 1, 2 and 3 were 62.3 % (52.4–72.3), 4.9 % (2.5–7.3) and 0.5 % (0.1–0.9) for ASAQ–FDC (from 32 sites); 58.4 % (50.2–66.6), 8.7 % (6.3–11.2) and 1.7 % (1.0–2.4) for ASAQ-loose NFDC (from 43 sites); and 58.9 % (52.6–65.3), 10.6 % (0–21.3) and 2.4 % (0–5.7) for ASAQ-coblistered NFDC (from 9 sites), respectively. Detailed information of PPR is presented in Additional file 4. ACT, artemisinin-based combination therapy; AL, artemether-lumefantrine; AS-AQ, artesunate-amodiaquine; DP, dihydroartemisinin-piperaquine; PPR, parasite positivity rate

**Table 3:** Study sites with day 3 parasite positivity rate (PPR) >3 %

Study site (country)	Year	Treatment	Day 3 PPR (95 % CI)^a^
New Halfa (Sudan)	2006	AL	3.0 % (1/33) (0.5–15.3)
ELWA Hospital (Liberia)	2007	AL	3.4 % (2/58) (0.9–11.7)
JFK Hospital (Liberia)	2007	AL	3.8 % (2/53) (1.0–12.8)
Bagamoyo (Tanzania)	2004	AL	4.0 % (2/50) (1.1–13.5)
Afokang (Nigeria)	2007–08	AL	5.9 % (10/170) (3.2–10.5)
Ndumo (South Africa)	2002	AL	6.0 % (6/100) (2.8–12.5)
San Pedro (Côte d’Ivoire)	2012	AL	6.5 % (2/31) (1.8–20.7)
Gedaref (Sudan)	2006	AL	7.8 % (4/51) (3.1–18.5)
Andapa (Madagascar)	2007	AS-AQ (loose NFDC)	3.3 % (1/30) (0.6–16.7)
Gaya (Niger)	2011	AS-AQ (FDC)	3.9 % (3/77) (1.3–10.8)
Grand Gedeh County (Liberia)	2010–11	AS-AQ (FDC)	3.9 % (4/102) (1.5–9.7)
Dabola (Guinea)	2004	AS-AQ (loose NFDC)	4.5 % (5/110) (1.9–10.2)
Afokang (Nigeria)	2007–08	AS-AQ (FDC)	5.2 % (9/173) (2.8–9.6)
Malakal (Sudan)	2003	AS-AQ (loose NFDC)	5.3 % (7/131) (2.6–10.6)
Kuito (Angola)	2003	AS-AQ (loose NFDC)	5.4 % (5/93) (2.3–11.9)
Kailahun (Sierra Leone)	2004	AS-AQ (loose NFDC)	5.6 % (7/125) (2.7–11.1)
Mlomp (Senegal)	1999	AS-AQ (loose NFDC)	5.8 % (9/154) (3.1–10.7)
Richard Toll (Senegal)	2003	AS-AQ (loose NFDC)	7.1 % (3/42) (2.5–19.0)
Miandrivazo (Madagascar)	2006	AS-AQ (loose NFDC)	10.3 % (7/68) (5.1–19.8)^b^
Yaoundé (Cameroon)	2005	AS-AQ (coblistered NFDC)	30.7 % (31/101) (22.5–40.3)^b^
Manhiça (Mozambique)	2005–06	DP	4.0 % (12/299) (2.3–6.9)
Afokang (Nigeria)	2007–08	DP	7.7 % (11/142) (4.4–13.3)

^a^Associated 95 % confidence interval computed using Wilson’s method; ^b^these sites have day 3 PPR >10 % and would be classed as sites with suspected partial artemisinin resistance requiring further investigation. Patients in Miandrivazo were treated with ASAQ-loose NFDC and those in Yaoundé treated with ASAQ-coblistered NFDC. AL, artemether-lumefantrine; AS-AQ, artesunate-amodiaquine; DP, dihydroartemisinin-piperaquine; NFDC, non-fixed dose combination; PPR, parasite positivity rate

### Risk factors associated with the parasite positivity status

**Fig. 3:**
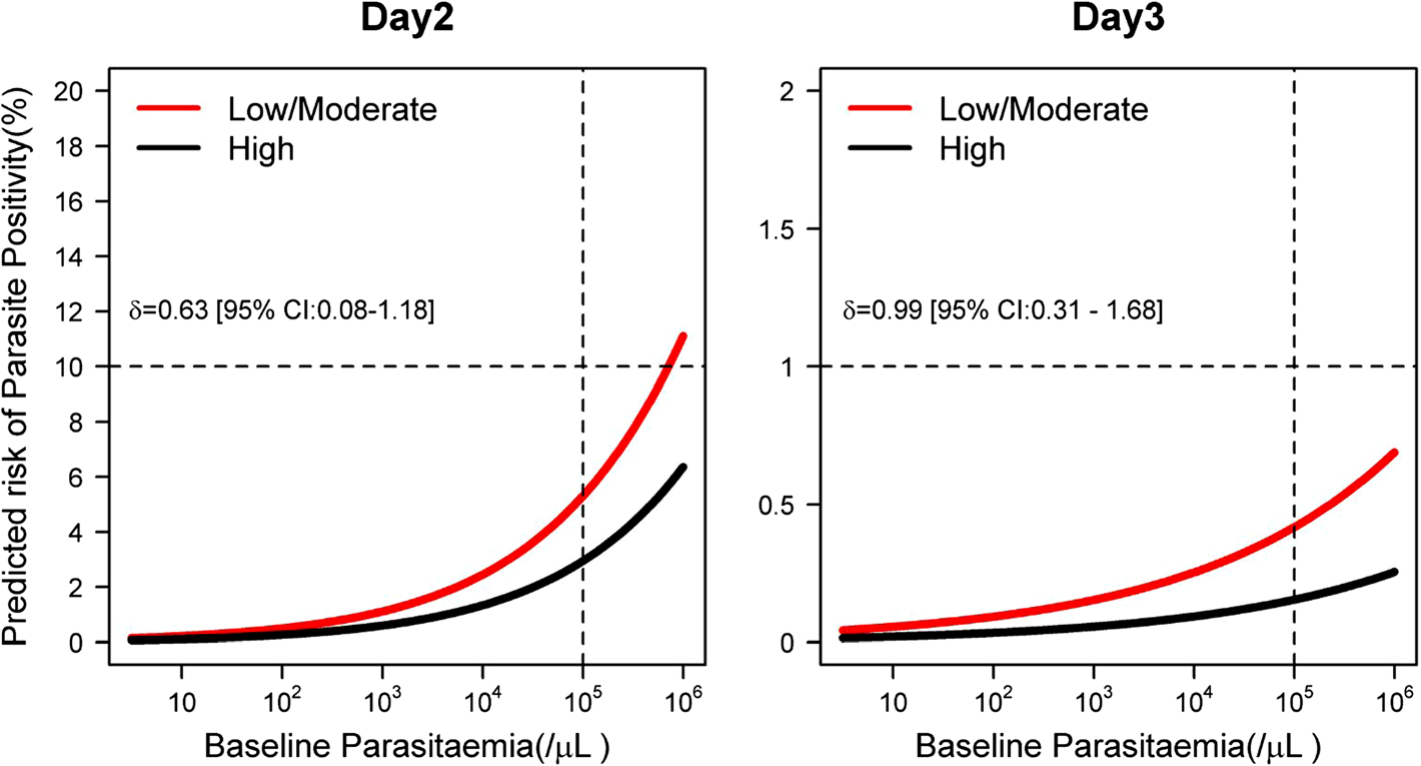
Probability of remaining parasitaemic (%) on days 2 and 3 for a given baseline parasitaemia in areas with different levels of transmission for children from 1 to 5 years of age. The probability of remaining positive on a given day was generated using coefficients from the final multivariable logistic regression with random effects for study sites. Zero study site effect was assumed for generating the predicted risk. The difference in risk of positivity for low/moderate setting has been given as *δ* and associated 95 % confidence interval presented

The independent risk factors for parasite positivity were similar on days 1 and 2 (see Additional file 4: Table S6 for details on day 1 and Table 4 for day 2). After adjusting for confounding factors, patients treated with AL were at an increased risk of remaining parasitaemic on day 2 (adjusted odds ratio (AOR) = 1.21 (95 % CI: 1.01–1.44), *P* = 0.040) compared to those treated with DP or those treated with ASAQ-FDC (AOR = 1.33 (95 % CI: 1.08–1.63), *P* = 0.005). Similarly, patients treated with ASAQ-loose NFDC had an increased risk of remaining parasitaemic on day 2 compared to DP (AOR = 1.46 (95 % CI: 1.05–2.01), *P* = 0.022) and compared to ASAQ-FDC (AOR = 1.61 (95 % CI: 1.14–2.29), *P* = 0.007). In the same multivariable model, patients from low/moderate transmission sites were also at greater risk of remaining parasitaemic on day 2 compared to those from high transmission sites (AOR = 1.88 (95 % CI: 1.09–3.24), *P* = 0.024) (Fig. 3).

**Table 4:** Univariable and multivariable risk factors for parasite positivity on day 2

			Univariable analysis		Multivariable analysis^c^	
Variable	N (n)^a^	Random effects^b^	Crude OR (95 % CI)	*P* value	Adjusted OR (95 % CI)	*P* value
Baseline parasitaemia (2-fold rise)	27,496 (1,853)	2.31	1.30 (1.26–1.34)	<0.001	1.27 (1.24–1.31)	<0.001
Baseline anaemia						
Non-anaemic (reference)^d^	8,838 (544)	2.14	1	-	-	-
Moderate	9,652 (714)		1.07 (0.94–1.22)	0.274	1.07 (0.94–1.22)	0.289
Severe	1,668 (124)		1.24 (0.99–1.55)	0.056	1.33 (1.06–1.67)	0.014
Unknown	7,338 (471)		-	-	-	-
Gametocytes presence						
No (reference)	18,672 (1,358)	2.08	1	-	-	-
Yes	1,979 (102)		0.95 (0.74–1.2)	0.650	-	-
Febrile on presentation (temperature >37.5 °C)						
No (reference)	9,355 (433)	2.06	1	-	-	-
Yes	17,217 (1,412)		1.72 (1.52–1.95)	<0.001	1.46 (1.28–1.66)	<0.001
Gender^e^						
Female (reference)	12,873 (835)	2.22	1	-	-	-
Male	13,995 (982)		1.11 (1.00–1.23)	0.052	-	-
Age category						
≥12 years (reference)	4,245 (202)	2.22	1	-	-	-
<1 year	2,014 (139)		1.89 (1.40–2.57)	<0.001	1.49 (1.09–2.05)	0.013
1 to <5 years	15,677 (1,176)		1.94 (1.52–2.46)	<0.001	1.54 (1.21–1.97)	0.001
5 to <12 years	5,528 (334)		1.49 (1.20–1.85)	<0.001	1.25 (1.00–1.56)	0.048
Transmission settings						
High (reference)	10,368 (455)	2.12	1	-	-	-
Low/moderate	17,128 (1,398)		1.50 (0.88–2.55)	0.135	1.88 (1.09–3.24)	0.024
Treatment^f^						
DP (reference)	4,420 (340)	2.12	1	-	-	-
AL	12,255 (729)		1.19 (1.00–1.42)	0.050	1.21 (1.01–1.44)	0.040
ASAQ-FDC	4,997 (246)		0.94 (0.75–1.19)	0.619	0.90 (0.71–1.14)	0.388
ASAQ-coblistered NFDC	1,574 (167)		1.80 (0.84–3.85)	0.130	1.87 (0.86–4.04)	0.113
ASAQ-loose NFDC	4,250 (371)		1.62 (1.18–2.22)	0.003	1.46 (1.05–2.01)	0.022

^a^N, number of patients with non-missing data; n, number of patients with positive blood smear on day 2; ^b^variance of the random effects for the univariable analyses; ^c^N = 26,544 for the final multivariable model with 1,843 cases of positive parasitaemia. Likelihood ratio test for random effect (*P* <0.001). Variance of random effect = 2.05. Proportion of total variance contributed by the site-level variance component (ρ) = 0.38. Coefficient (standard error) of intercept = −7.95 (0.3539). The coefficient of variation in parameter estimates was calculated by excluding one study site at a time and expressed as relative standard deviation (RSD). Distributions of the adjusted odds ratio (AOR) were generated from 250 bootstrap samples. The RSD and bootstrap distribution are shown in Additional file 4: Table S8 and Figure S3); ^d^multiple imputation was performed on missing anaemia status using ordinal logistic regression with age, gender and parasitaemia as covariates. The estimates derived using 100 imputations for moderate and severe anaemia are: AOR = 1.05 (95 % CI: 0.93–1.19), *P* = 0.446; and AOR = 1.24 (95 % CI: 0.99–1.55), *P* = 0.056, respectively; ^e^gender (AOR = 1.10 (95 % CI: 0.99–1.22), *P* = 0.079 using likelihood ratio test) was no longer significant in the presence of the other variables shown in the multivariable model and hence dropped; ^f^for AL compared to ASAQ-FDC (AOR = 1.33 (95 % CI: 1.08–1.63, *P* = 0.005). For ASAQ-loose NFDC compared to ASAQ-FDC (AOR = 1.61 (95 % CI: 1.14–2.29), *P* = 0.007). AL, artemether-lumefantrine; AS-AQ, artesunate-amodiaquine; ASAQ-coblistered NFDC, non-fixed dose combination in a co-blister formulation; ASAQ-FDC, fixed dose combination; ASAQ-loose NFDC, non-fixed dose combination in a loose formulation; DP, dihydroartemisinin-piperaquine

In multivariable analysis, the risk of being parasitaemic on day 3 increased with baseline parasitaemia (AOR = 1.16 (95 % CI: 1.08–1.25), for every 2-fold increase in parasite density, *P* <0.001), fever (AOR = 1.50 (95 % CI: 1.06–2.13), *P* = 0.022), severe anaemia (Hb < 7 g/dl) (AOR = 2.04 (95 % CI: 1.21–3.44), *P* = 0.008) and being from areas of low/moderate transmission (AOR = 2.71 (95 % CI: 1.38–5.36, *P* = 0.004 compared to high transmission areas); see Table 5. Patients treated with ASAQ-loose NFDC were at 2.27-fold ((95 % CI: 1.14–4.51), *P* = 0.020) increased risk of being parasitaemic on day 3 compared to patients treated with DP and 3.36-fold ((95 % CI: 1.61–6.98), *P* = 0.001) higher risk compared to patients treated with ASAQ-FDC. Similarly, patients treated with ASAQ-coblistered NFDC were at 4.18-fold ((95 % CI: 1.28–13.68), *P* = 0.017) greater risk compared to those treated with ASAQ-FDC (Table 5).

**Table 5:** Univariable and multivariable risk factors for parasite positivity on day 3

			Univariable analysis		Multivariable analysis^c^	
Variable	N (n)^a^	Random effects^b^	Crude OR (95 % CI)	*P* value	Adjusted OR (95 % CI)	*P* value
Baseline parasitaemia (2-fold rise)	28,580 (253)	2.57	1.18 (1.10–1.28)	<0.001	1.16 (1.08–1.25)	<0.001
Baseline anaemia						
Non-anaemic (reference)^d^	9,368 (60)	2.50	1	-	-	-
Moderate	9,926 (86)		1.14 (0.80–1.61)	0.473	1.14 (0.80–1.61)	0.476
Severe	1,697 (23)		1.94 (1.15–3.25)	0.012	2.04 (1.21–3.44)	0.008
Unknown	7,589 (84)		1.08 (0.55–2.13)	0.827	-	-
Gametocytes presence						
No (reference)	19,561 (168)	3.20	1	-	-	-
Yes	2,038 (17)		1.10 (0.63–1.91)	0.747	-	-
Febrile on presentation (temperature >37.5 °C)						
No (reference)	9,874 (46)	2.27	1	-	-	-
Yes	17,678 (207)		1.68 (1.19–2.38)	0.003	1.50 (1.06–2.13)	0.022
Gender						
Female (reference)	13,439 (106)	2.56	1	-	-	-
Male	14,511 (142)		1.22 (0.94–1.58)	0.134	-	-
Age category						
≥12 years (reference)	4,639 (36)	2.55	1	-	-	-
<1 year	2,027 (20)		1.51 (0.75–3.03)	0.247	1.25 (0.62–2.55)	0.530
1 to <5 years	16,060 (130)		1.23 (0.72–2.10)	0.453	1.09 (0.64–1.87)	0.753
5 to <12 years	5,818 (66)		1.74 (1.09–2.76)	0.019	1.56 (0.98–2.48)	0.061
Transmission settings						
High (reference)	10,377 (66)	2.38	1	-	-	-
Low/moderate	18,203 (187)		2.34 (1.14–4.80)	0.021	2.71 (1.38–5.36)	0.004
Treatment^e^						
DP (reference)	4,434 (34)	2.01	1	-	-	-
AL	13,004 (76)		0.93 (0.57–1.51)	0.765	0.93 (0.57–1.52)	0.774
ASAQ-FDC	4,999 (27)		0.70 (0.38–1.31)	0.269	0.67 (0.36–1.25)	0.206
ASAQ-coblistered NFDC	1,851 (44)		2.23 (0.69–7.22)	0.183	2.87 (0.89–9.27)	0.078
ASAQ-loose NFDC	4,292 (72)		2.27 (1.12–4.60)	0.023	2.27 (1.14–4.51)	0.020

^a^N = number of patients with non-missing data; n = number of patients with positive blood smear on day 3; ^b^variance of the random effects for the respective univariable analyses; ^c^N = 27,520 for the final multivariable model with 252 cases of positive parasitaemia. Likelihood ratio test for random effect (*P* <0.001). Variance of random effect = 1.72. Proportion of total variance contributed by the site-level variance component (ρ) = 0.35. Coefficient (standard error) of intercept = −9.07 (0.7084). The coefficient of variation in parameter estimates was calculated by excluding one study site at a time and expressed as relative standard deviation (RSD). The RSD is shown in Additional file 4: Table S9; ^d^multiple imputation was performed on missing anaemia status using ordinal logistic regression with age, gender and parasitaemia as covariates. The estimates derived using 100 imputations for moderate and severe anaemia are: AOR = 1.11 (95 % CI: 0.80–1.54), *P* = 0.523 and AOR = 1.62 (95 % CI: 0.99–2.66), *P* = 0.057, respectively; ^e^for ASAQ-loose NFDC: AOR = 2.27 (95 % CI: 1.14–4.51), *P* = 0.020 compared to DP and AOR = 3.36 (95 % CI: 1.61–6.98), *P* = 0.001 compared to ASAQ-FDC. For ASAQ-coblistered NFDC, AOR = 4.18 (95 % CI: 1.28–13.68), *P* = 0.017 compared to ASAQ-FDC. AL, artemether-lumefantrine; AS-AQ, artesunate-amodiaquine; ASAQ-coblistered NFDC, non-fixed dose combination in a co-blister formulation; ASAQ-FDC, fixed dose combination; ASAQ-loose NFDC, non-fixed dose combination in a loose formulation; DP, dihydroartemisinin-piperaquine

### Effect of weight adjusted (mg/kg) artemisinin components

The weight adjusted drug dosage (mg/kg) was available in 72 % (21,310/29,493) of the patients. Adjusted for the baseline confounders, the mg/kg dose of artemisinin component was not associated with the risk of parasite positivity on any day for patients treated with DP or AS-AQ (either for the fixed or the loose combinations). However, in patients treated with AL, an increased mg/kg dose of artemether was associated with a lower risk of patent parasitaemia only on day 1. Every unit increase in daily mg/kg artemether dose reduced the risk of parasite positivity by 5 % ((95 % CI: 1–7 %), *P* = 0.003) (see Additional file 4: Table S10).

### Derivation of day 3 PPR threshold for suspected diminished artemisinin susceptibility

**Fig. 4:**
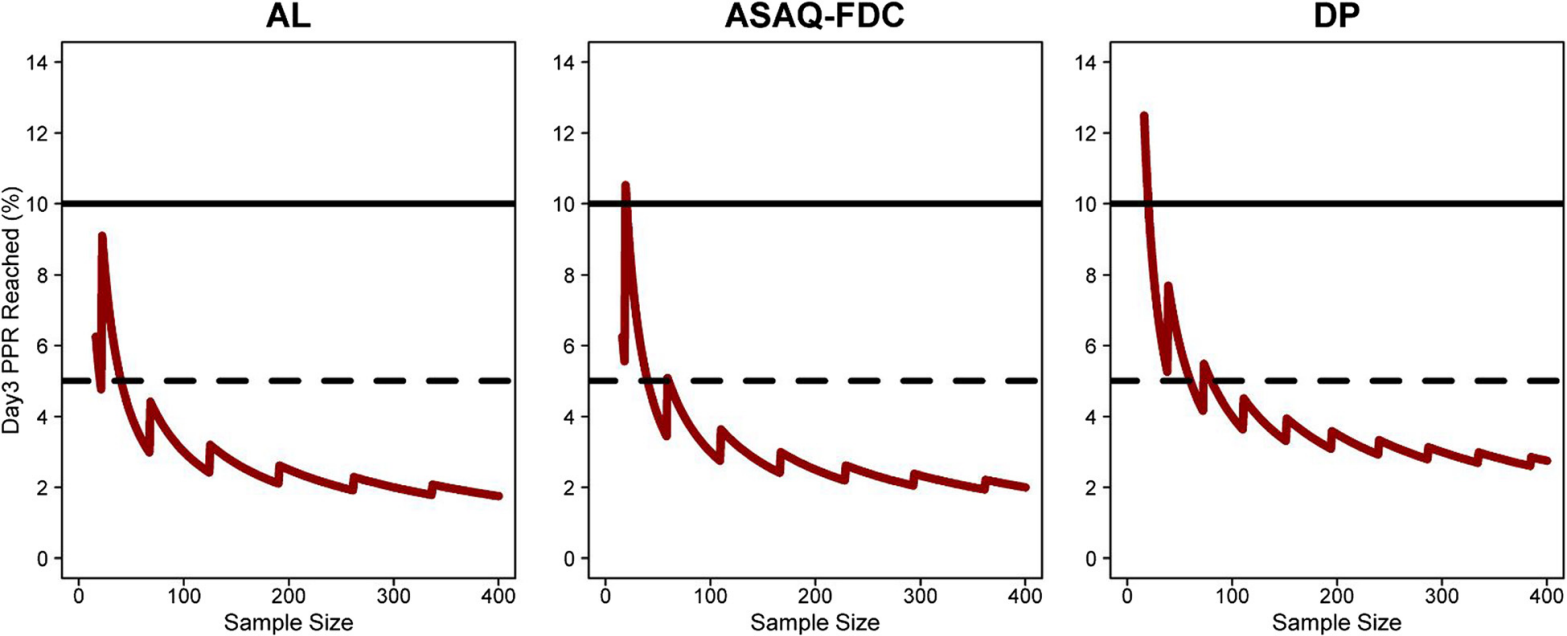
Maximum day 3 parasite positivity rate (PPR) possible for each of the treatment regimens for a given study sample size. Worst-case estimates were used for the analysis, that is, an upper limit of 95 % CI was assumed to be the true underlying parasite positivity rate on day 3, which was 0.82 %, 0.94 % and 1.42 % for AL, ASAQ-FDC and DP, respectively. The horizontal solid line represents 10 % day 3 WHO threshold and the dotted horizontal line represents 5 % day 3 PPR. The saw-tooth spikes are the result of rounding to the nearest whole number. ACT, artemisinin-based combination therapy; AL, artemether-lumefantrine; ASAQ-FDC, fixed dose combination; DP, dihydroartemisinin-piperaquine; PPR, parasite positivity rate

The overall day 3 PPR was 0.58 % (95 % CI: 0.34–0.82) for AL, 0.54 % (95 % CI: 0.14–0.94) for ASAQ-FDC and 0.77 % (95 % CI: 0.11–1.42) for DP. In studies with a sample size greater than 50 patients, the observed PPR was unlikely to exceed 5 % positivity on day 3 (Fig. 4). However, in studies with fewer than 50 patients, the variance around the estimate was extremely wide, so a reliable estimate could not be derived (Table 6, Fig. 4).

**Table 6:** Upper limit of parasite positivity rates (PPRs) which could be observed on day 3

Variable	AL		ASAQ-FDC		DP	
	Day 3 PPR (95 % CI)	Maximum predicted risk^a^	Day 3 PPR (95 % CI)	Maximum predicted risk^a^	Day 3 PPR (95 % CI)	Maximum predicted risk^a^
Age category						
<1 year	0.79 (0.00–1.67)	6.50	0.67 (0.00–2.00)	7.69	0.68 (0.00–1.45)	6.00
1 to <5 years	0.54 (0.28–0.79)	4.30	0.70 (0.19–1.21)	6.00	0.83 (0.00–1.65)	6.35
5 to <12 years	0.69 (0.26–1.11)	6.00	0.32 (0.00–0.76)	4.11	0.70 (0.07–1.34)	6.00
≥12 years	0.49 (0.11–0.88)	4.76	0.13 (0.00–0.40)	4.00	0.48 (0.00–1.06)	5.66
Transmission						
High	0.20 (0.03–0.37)	4.00	0.27 (0.06–0.48)	4.00	0.05 (0.00–0.15)	2.00
Low/moderate	0.79 (0.43–1.15)	6.00	0.65 (0.09–1.22)	6.00	1.28 (0.27–2.29)	8.00
Parasitaemia (x 1,000 parasites/μl)						
<10	0.39 (0.18–0.60)	4.00	0.43 (0.00–0.91)	4.92	0.87 (0.00–1.81)	7.02
10 to <50	0.58 (0.30–0.86)	4.62	0.35 (0.00–0.72)	4.00	0.54 (0.01–1.08)	5.77
50 to <100	0.81 (0.32–1.30)	6.00	1.01 (0.20–1.83)	7.02	0.86 (0.00–1.85)	7.14
≥100	1.04 (0.35–1.74)	6.67	1.12 (0.00–2.25)	8.00	1.15 (0.13–2.17)	8.00
Study sample size						
<50	1.02 (0.12–1.92)	10.34	0.78 (0.00–2.00)	10.71	0.00 (0.00–7.71)	20.68
50 to <100	0.72 (0.30–1.13)	6.00	0.55 (0.00–1.53)	6.00	0.25 (0.00–0.50)	4.00
100 to <200	0.90 (0.34–1.45)	4.62	0.88 (0.08–1.68)	5.00	1.43 (0.00–3.03)	7.01
≥200	0.25 (0.05–0.45)	1.75	0.18 (0.02–0.35)	1.50	0.70 (0.00–1.73)	4.00
Overall	0.58 (0.34–0.82)	4.41	0.54 (0.14–0.94)	5.08	0.77 (0.11–1.42)	6.00

^a^The maximum predicted risk is the day 3 PPR which could be observed assuming the worst case day 3 PPR, that is, the upper limit of day 3 PPR 95 % CI. For calculating the maximum predicted risk for age, transmission and parasitaemia, a minimum study sample size of 50 in a study was assumed. AL, artemether-lumefantrine; ASAQ-FDC, fixed dose combination; DP, dihydroartemisinin-piperaquine; PPR, parasite positivity rate

### Assessment of potential bias

Attrition biases of the included studies are presented in Additional file 1. Sensitivity analyses showed that exclusion of any of the studies did not change the main conclusions of the analysis (Additional file 4: Table S12). In addition, parameter estimates obtained from bootstrap sampling were similar to the estimates from final multivariable models (Additional file 4: Figures S2,3). Combining studies with and without individual patient data concluded similar results to those in which only studies with individual patient data were available (Additional file 4: Table S13). Funnel plots of the log-transformed odds ratio against standard error were symmetric suggesting low risk of publication bias (Additional file 4: Figures S7,8).

## Discussion

This large pooled analysis of nearly 30,000 patients from trials conducted before 2012 highlights that parasite clearance after treatment with an ACT is still extremely rapid in Sub-Saharan Africa. More than 90 % of the patients were aparasitaemic by day 2 and 99 % by day 3, consistent with previous reports demonstrating rapid parasite clearance after treatment with ACTs in high transmission settings [20, 26].

In areas of intense transmission, immunity develops at a relatively young age [38, 39] and is a key determinant of the antimalarial therapeutic response [40]. Our results show that patients from areas of low/moderate transmission were at greater risk of parasite positivity compared to patients from high transmission regions, a likely reflection of the influence of immunity in the early therapeutic response. Almost 80 % of patients were less than 12 years old, an age group with the highest risk of parasitaemia on days 1 and 2. Every 2-fold increase in parasite density was associated with 1.5 to 1.2-fold risk of failing to clear parasitaemia on days 1 to 3, respectively. Similarly, patients with fever at enrolment had a higher risk of persistent parasitaemia. Fever and parasitaemia are closely correlated, with symptoms manifesting in those exceeding a pyrogenic threshold, this threshold rising as the host experiences repeated infections and acquires a degree of immunity. However, independent of baseline parasitaemia, patients with fever on presentation showed slower parasitological clearance as has been noted previously and hypothesized to relate to a reduced host immunity [25, 27]. The results of these analyses emphasize the importance of transmission intensity in the development of immunity and the pivotal role of acquired immunity in modulating early parasitological response to treatment with ACTs [22, 23]. Patients who were severely anaemic at presentation were also at greater risk of remaining parasitaemic on days 1 to 3 compared to those who were non-anaemic. Severe anaemia is associated with recurrent episodes of malaria and can arise as a consequence of treatment failure, hence may be indicative of a poor immune response or emerging parasite resistance [41]. In addition, co-infections with helminths, poor socioeconomic status and malnutrition may further compound the effects [42]. Further research is needed to understand the underlying biological pathways and will be explored in the WWARN Haematology Study Group [43].

After adjusting for these parasite and host factors, the risks of persistent parasitaemia on days 1 and 2 were higher in patients treated with AL compared to those treated with DP and ASAQ-FDC, but this difference was no longer apparent by day 3. Artemether is a lipophilic compound and is more slowly absorbed than artesunate or dihydroartemisinin, and this difference may explain the slower action of AL [44, 45]. Moreover, artemether is delivered in a lower dose which is split into twice daily target dosing of 1.7 mg/kg compared with the once daily dose of 4 mg/kg dose of dihydroartemisinin in DP and 4 mg/kg dose of artesunate in AS-AQ [46, 47]. This dose effect was apparent on day 1 but not on days 2 and 3, with every unit increase in artemether dose reducing the risk of day 1 positivity by 5 %, a result observed previously in a large pooled analysis [48]. Similarly, patients treated with ASAQ-loose NFDC were at increased risk of slow clearance on days 2 and 3 compared to those treated with ASAQ-FDC (and DP) despite the target dose of artesunate being the same (4 mg/kg/day) across all the formulations. The differences in the mg/kg amodiaquine dosage between different formulations were found not to affect early parasitological responses (data not shown). The elevated risk observed with the NFDCs could be associated with several factors including drug quality and tablet splitting required for many children, which could potentially lead to dosing inaccuracy or reduced compliance [49, 50].

The study period encompasses 1999 to 2012, covering the period during the introduction of the large scale deployment of ACTs across Africa. Overall, there were no differences in the early parasitological response post-ACT treatment in different sub-regions of SSA and there was no evidence of decreased susceptibility to artemisinin in Africa over this time period. Nevertheless, there were 22 sites where PPR on day 3 exceeded 3 % (the threshold below which artemisinin resistance in unlikely), with two sites exceeding day 3 PPR of 10 % (the WHO threshold for suspected partial resistance). In Miandrivazo (Madagascar), the reported PPR was 10.3 % in 2006 [51] but less than 1 % in a subsequent trial in the same region (Tsiroanomandidy) [52]. In Yaoundé, a PPR of 30 % was reported in 2005 [53]; however, in a study conducted at the same site 7 years later [54], the PPR was 2.9 % (95 % CI: 3.7–27.2, 2/68) suggesting that the high PPR observed in our dataset could have been an artefact. High day 3 PPR does not necessarily relate to a change in parasite susceptibility to artemisinin; other factors, such as declining immunity [55], poor drug quality [56] and variable quality of microscopy [57] can play major roles. Studies with more intense blood sampling are needed in areas of delayed parasite clearance [10, 12]. These will require better definition of the parasite clearance, complementary *in vitro* testing [58] and molecular analysis [13] to rule out any change in artemisinin susceptibility.

Our analysis has a number of limitations. First, the literature search was limited to prospective clinical trials indexed in PubMed and some relevant studies may have been overlooked. However, we actively looked for relevant trials (unpublished) and the research groups contacted represent the majority of the malaria community, which is relatively small and highly interactive. It is highly unlikely that any studies were missed. The assessment of publication bias (PB) showed that effect sizes were symmetrical suggesting low risk of bias in studies included. Of the 140 trials identified, individual patient data were available for inclusion for 71 of the published studies (50.7 %). To address this potential bias, included studies were compared with the published studies that were not available. There were no apparent differences in patient population and/or outcomes between the studies included and those where individual patient data were not available. Reassuringly, the results from two-stage meta-analyses, which combined studies with and without individual patient data, were also similar to the results obtained from studies where only individual patient data were available, suggesting that systematic attrition bias was unlikely. A second issue is that, although the days of follow-up were recorded in the studies, the actual time of blood collection was not. Daily samples were taken over a range of times and the interval between days is likely to have varied significantly from the desired 24-, 48- or 72-hour timelines. Third, the data used rely on quantitative microscopy and quality control on microscopy procedures were reported in only 60 % of the studies. Accurate recording of the time of sampling, harmonizing microscopy procedures and appropriate quality control procedures could greatly improve the precision of the parasite clearance time [11]. To facilitate this process, a new microscopy procedure has been developed recently to improve comparability of results between groups [59]. Finally, no data on drug levels were available to assess whether patients achieved therapeutic blood concentrations. However, absorption of artemisinin derivatives in uncomplicated malaria is usually good and in the majority (89 %) of studies, drug administration was observed fully or partially by the clinical team.

This large dataset provided a unique opportunity to identify a threshold for day 3 parasite positivity based upon African studies, below which artemisinin resistance is highly unlikely. The upper limit of the 95 % CI for day 3 PPR, indicative of the worst-case scenario, defines maximum PPR which could be observed reliably in a clinical trial. This threshold was vulnerable to the initial parasitaemia and study sample size. For example, in studies with 50 or less patients, the confidence interval around any threshold value was wide, hence its predictive utility under those circumstances is limited. Our results demonstrate that the 95th percentile of the observed day 3 PPR in Africa was 5.3 %, substantially lower than the currently recommended threshold of 10 % for suspected partial artemisinin resistance. These findings strongly suggest that a ‘one size fits all’ threshold of 10 % should be used with caution. A simple sensitive parameter indicative of potential artemisinin resistance would be an extremely useful surveillance tool. Our analysis suggests that although the widely proposed 10 % threshold would be specific, it lacks sensitivity in detecting an early stage changes of delayed parasite clearance. Moreover, a previous WWARN meta-analysis of published literature showed that the PPR on day 3 over the same period (1999–2012) was much lower in Africa (1 %) compared to Asia (3.8 %) [26]. A threshold of 5 % provides greater sensitivity and an early warning signal in SSA. Modelling will help to refine this threshold further [21, 60].

## Conclusion

In conclusion, this pooled analysis provides critical baseline information regarding early parasitological response post-treatment with ACTs in SSA. The assessment of the host, parasite and drug determinants which influence the early parasitological response can provide evidence-based guidance for monitoring the early signs of artemisinin resistance and effective case management that will be critical in optimizing malaria control and containment efforts.

## Additional files

Additional file 1:
**References of all clinical trials and their study designs.** (XLSX 1094 kb) Additional file 2:
**Maps showing locations of published clinical efficacy studies and the studies included in the pooled analysis.** (PDF 173 kb) Additional file 3:
**Transmission classification.** (XLSX 29 kb) Additional file 4:
**Additional tables and figures.** (DOCX 379 kb) Additional file 5:
**Authors and contributions.** (XLS 82 kb)

## Abbreviations

ACT: Artemisinin-based combination therapy
AL: Artemether-lumefantrine
AOR: Adjusted odds ratio
AQ: Amodiaquine
AS: Artesunate
AS-AQ: Artesunate-amodiaquine
ASAQ-coblistered NFDC: Non-fixed dose combination in a co-blister formulation
ASAQ-FDC: Fixed dose combination
ASAQ-loose NFDC: Non-fixed dose combination in a loose formulation
CI: Confidence interval
DP: Dihydroartemisinin-piperaquine
IPD: Individual participant data
IQR: Interquartile range
LLIN: Long-lasting insecticidal net
OR: Odds ratio
OxTREC: Oxford Tropical Research Ethics Committee
PPR: Parasite positivity rate
PRISMA: Preferred Reporting Items for Systematic Reviews and Meta-Analyses
RSD: Relative standard deviation
SD: Standard deviation
SSA: Sub-Saharan Africa
TDR: The Special Programme for Research and Training in Tropical Diseases
WHO: World Health Organization
WWARN: Worldwide Antimalarial Resistance Network
