# Supplementary material for: Clinical determinants of early parasitological response to ACTs in African patients with uncomplicated falciparum malaria: a literature review and meta-analysis of individual patient data
Source: BMC Med. 2015 Sep 7;13:212. doi: 10.1186/s12916-015-0445-x (PMC4561425; doi:10.1186/s12916-015-0445-x)
Supplement: Additional file 2: — Maps showing locations of published clinical efficacy studies and the studies included in the pooled analysis. (PDF 173 kb) [file 12916_2015_445_MOESM2_ESM.pdf]

## Study sites for clinical trials included in the WWARN ACT Africa Baseline Pooled Analysis

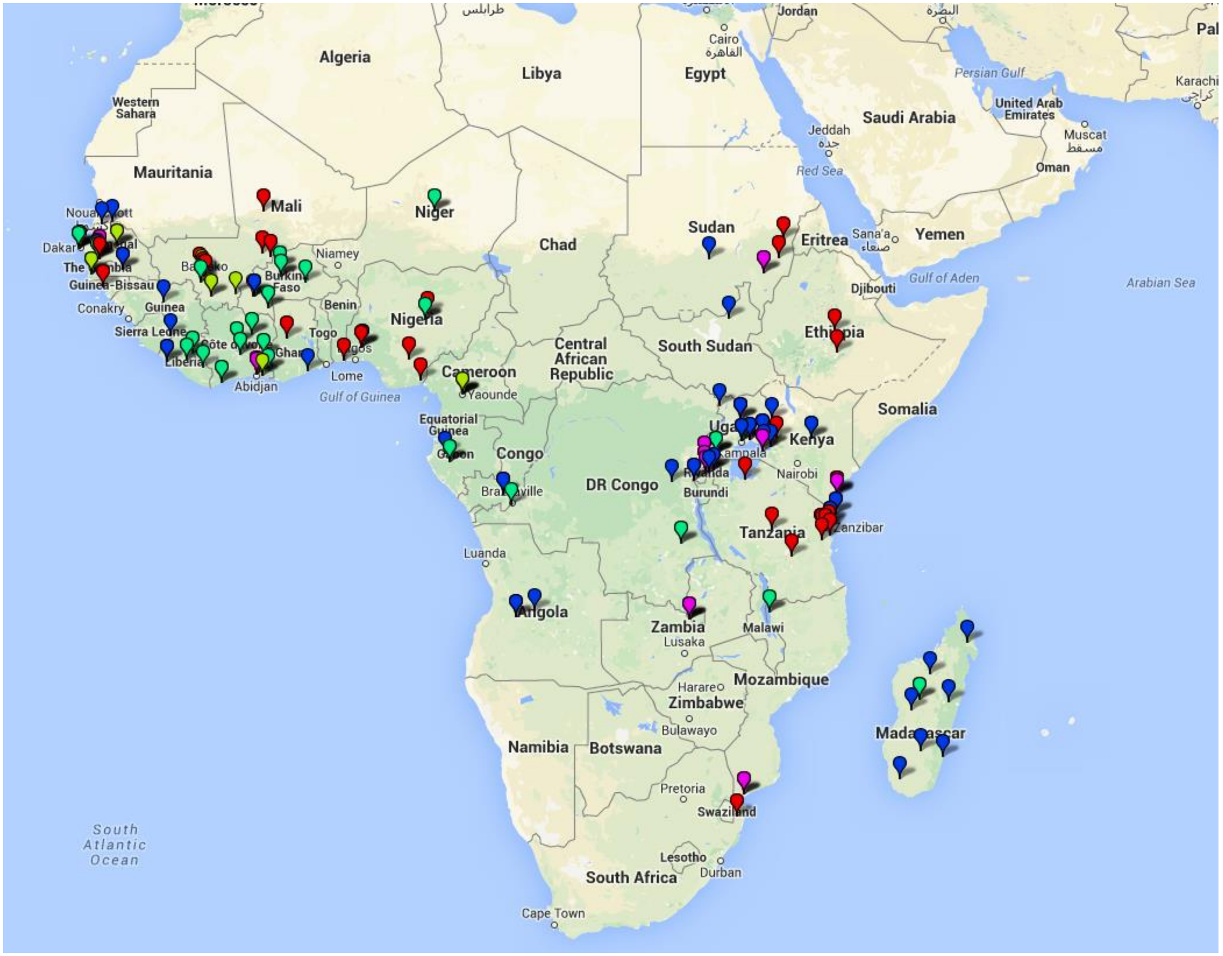


**Legend:** Coblistered Artesuante-Amodiaquine (ASAQ-Co-blisters), ASAQ Fixed Dose Combination (ASAQ-FDC), ASAQ- Loose formulation (ASAQ-Loose), Dihydroartemisinin Piperaquine (DP), Artemether Lumefantrine (AL)
