# Supplementary material for: Clinical determinants of early parasitological response to ACTs in African patients with uncomplicated falciparum malaria: a literature review and meta-analysis of individual patient data
Source: BMC Med. 2015 Sep 7;13:212. doi: 10.1186/s12916-015-0445-x (PMC4561425; doi:10.1186/s12916-015-0445-x)
Supplement: Additional file 4: — Additional tables and figures. (DOCX 379 kb) [file 12916_2015_445_MOESM4_ESM.docx]

**Supplementary File-4: Additional tables and figures**

**Supplementary Table 1: Early parasitological response for AL by country and year of the study**

| **Country** | **2002** | **2003** | **2004** | **2005** | **2006** | **2007** | **2008** | **2009** | **2010** | **2011** | **2012** |
| --- | --- | --- | --- | --- | --- | --- | --- | --- | --- | --- | --- |
| **Angola** |  |  |  |  |  |  |  |  |  |  |  |
| Day1 | - | - | - | - | - | - | - | - | - | - | - |
| Day2 | - | - | 2[1/64] | - | - | - | - | - | - | - | - |
| Day3 | - | - | 0[0/64] | - | - | - | - | - | - | - | - |
| **Benin** |  |  |  |  |  |  |  |  |  |  |  |
| Day1 | - | - | - | - | 56[24/43] | 0[0/24] | - | - | - | - | - |
| Day2 | - | - | - | - | 0[0/81] | 0[0/28] | - | - | - | - | - |
| Day3 | - | - | - | - | 0[0/81] | 0[0/28] | - | - | - | - | - |
| **Burkina Faso** |  |  |  |  |  |  |  |  |  |  |  |
| Day1 | - | - | - | 59[58/99] | - | 62[57/92] | 67[134/201] | - | - | 0[0/6] | - |
| Day2 | - | - | - | 4[14/358] | 2[5/241] | 0[0/91] | 0[0/373] | 0[0/74] | 3[7/231] | 1[1/131] | - |
| Day3 | - | - | - | 1[4/358] | 0[0/241] | 0[0/92] | 0[0/373] | 0[0/74] | 0[0/231] | 0[0/133] | - |
| **Cameroon** |  |  |  |  |  |  |  |  |  |  |  |
| Day1 | - | - | - | - | 70[39/56] | 65[31/48] | 72[18/25] | 80[20/25] | - | - | - |
| Day2 | - | - | - | - | 4[2/54] | 4[2/48] | 24[6/25] | 32[8/25] | - | - | - |
| Day3 | - | - | - | - | 0[0/54] | 0[0/48] | 0[0/25] | 0[0/25] | - | - | - |
| **Congo** |  |  |  |  |  |  |  |  |  |  |  |
| Day1 | - | - | - | - | - | - | - | - | - | - | - |
| Day2 | - | - | 2[2/105] | - | - | - | - | - | - | - | - |
| Day3 | - | - | 0[0/106] | - | - | - | - | - | - | - | - |
| **DRC** |  |  |  |  |  |  |  |  |  |  |  |
| Day1 | - | - | - | - | - | - | 20[1/5] | - | - | - | - |
| Day2 | - | - | - | - | - | - | 6[7/121] | 0[0/22] | - | - | - |
| Day3 | - | - | - | - | - | - | 1[1/121] | 0[0/22] | - | - | - |
| **Ethiopia** |  |  |  |  |  |  |  |  |  |  |  |
| Day1 | - | - | - | - | - | - | - | - | - | - | - |
| Day2 | - | - | - | - | - | - | 4[3/74] | 3[11/377] | - | - | - |
| Day3 | - | - | - | - | - | - | 0[0/74] | 1[4/376] | - | - | - |
| **Gabon** |  |  |  |  |  |  |  |  |  |  |  |
| Day1 | - | - | - | - | - | 29[2/7] | 65[45/69] | - | - | - | - |
| Day2 | - | - | - | - | - | 0[0/7] | 3[2/69] | - | - | - | - |
| Day3 | - | - | - | - | - | 0[0/7] | 0[0/69] | - | - | - | - |
| **Ghana** |  |  |  |  |  |  |  |  |  |  |  |
| Day1 | - | - | - | - | - | 29[8/28] | - | - | - | - | - |
| Day2 | - | - | - | - | - | 0[0/20] | - | - | - | - | - |
| Day3 | - | - | - | - | - | 0[0/28] | - | - | - | - | - |
| **Guinea-Bissau** |  |  |  |  |  |  |  |  |  |  |  |
| Day1 | - | - | - | - | 93[26/28] | 87[46/53] | 78[83/107] | - | - | - | - |
| Day2 | - | - | - | - | 50[14/28] | 28[15/53] | 26[28/106] | - | - | - | - |
| Day3 | - | - | - | - | 11[3/28] | 0[0/53] | 1[1/106] | - | - | - | - |
| **Côte d'Ivoire** |  |  |  |  |  |  |  |  |  |  |  |
| Day1 | - | - | - | 7[8/115] | 38[81/212] | - | 31[61/196] | 0[0/3] | - | - | 96[114/119] |
| Day2 | - | - | - | 4[5/115] | 3[7/211] | 0[0/75] | 2[4/196] | 0[0/3] | - | - | 19[21/113] |
| Day3 | - | - | - | 1[1/115] | 0[0/211] | 0[0/75] | 1[2/196] | 0[0/3] | - | - | 2[2/112] |
| **Kenya** |  |  |  |  |  |  |  |  |  |  |  |
| Day1 | 62[61/99] | 67[4/6] | 71[110/156] | 89[238/266] | 61[61/100] | 70[318/453] | - | - | - | - | - |
| Day2 | 0[0/96] | 0[0/6] | 4[7/156] | 12[31/266] | 0[0/90] | 21[90/435] | - | 3[5/150] | - | - | - |
| Day3 | 0[0/97] | 0[0/6] | 1[1/156] | 0[0/266] | 0[0/100] | 1[3/455] | - | 0[0/152] | - | - | - |
| **Liberia** |  |  |  |  |  |  |  |  |  |  |  |
| Day1 | - | - | - | - | - | 4[4/111] | - | - | - | - | - |
| Day2 | - | - | - | - | - | 13[14/110] | 4[9/235] | 1[6/401] | - | - | - |
| Day3 | - | - | - | - | - | 4[4/111] | 0[1/227] | 0[1/398] | - | - | - |
| **Madagascar** |  |  |  |  |  |  |  |  |  |  |  |
| Day1 | - | - | - | - | 55[33/60] | - | - | - | - | - | - |
| Day2 | - | - | - | - | 2[1/60] | - | - | - | - | - | - |
| Day3 | - | - | - | - | 0[0/60] | - | - | - | - | - | - |
| **Mali** |  |  |  |  |  |  |  |  |  |  |  |
| Day1 | - | - | 68[160/235] | - | 75[187/249] | 64[14/22] | - | 76[250/331] | - | - | - |
| Day2 | - | 1[1/194] | 4[12/341] | 2[3/129] | 4[14/362] | 0[0/22] | - | 5[18/328] | - | - | - |
| Day3 | - | 0[0/194] | 0[0/341] | 1[1/129] | 1[2/366] | 0[0/22] | - | 0[0/329] | - | - | - |
| **Mozambique** |  |  |  |  |  |  |  |  |  |  |  |
| Day1 | - | - | - | 77[27/35] | 92[158/172] | - | - | - | - | - | - |
| Day2 | - | - | - | 31[11/35] | 23[41/175] | - | - | - | - | - | - |
| Day3 | - | - | - | 0[0/35] | 1[2/178] | - | - | - | - | - | - |
| **Niger** |  |  |  |  |  |  |  |  |  |  |  |
| Day1 | - | - | - | - | - | - | - | - | - | 75[53/71] | - |
| Day2 | - | - | - | - | - | - | - | - | - | 14[10/70] | - |
| Day3 | - | - | - | - | - | - | - | - | - | 3[2/72] | - |
| **Nigeria** |  |  |  |  |  |  |  |  |  |  |  |
| Day1 | 22[21/97] | 100[3/3] | - | - | - | 32[43/133] | 92[156/170] | - | - | - | - |
| Day2 | 0[0/97] | 0[0/3] | - | - | - | 18[16/89] | 40[68/169] | - | - | - | - |
| Day3 | 0[0/99] | 0[0/3] | - | - | - | 2[3/131] | 5[8/171] | - | - | - | - |
| **Rwanda** |  |  |  |  |  |  |  |  |  |  |  |
| Day1 | - | - | - | - | - | 67[18/27] | 45[54/120] | - | - | - | - |
| Day2 | - | - | - | - | - | 7[2/27] | 2[2/120] | - | - | - | - |
| Day3 | - | - | - | - | - | 0[0/27] | 0[0/120] | - | - | - | - |
| **Senegal** |  |  |  |  |  |  |  |  |  |  |  |
| Day1 | - | 25[7/28] | - | - | 55[139/251] | 67[111/165] | 47[130/277] | - | 58[64/110] | 50[81/163] | - |
| Day2 | - | 4[1/28] | - | - | 5[12/250] | 4[7/165] | 6[17/277] | - | 4[9/247] | 6[9/162] | - |
| Day3 | - | 0[0/28] | - | - | 1[3/250] | 0[0/165] | 1[4/277] | - | 0[0/247] | 2[3/163] | - |
| **South Africa** |  |  |  |  |  |  |  |  |  |  |  |
| Day1 | 79[79/100] | - | - | - | - | - | - | - | - | - | - |
| Day2 | 25[25/100] | - | - | - | - | - | - | - | - | - | - |
| Day3 | 6[6/100] | - | - | - | - | - | - | - | - | - | - |
| **Sudan** |  |  |  |  |  |  |  |  |  |  |  |
| Day1 | - | - | - | - | 30[24/81] | - | - | 20[5/25] | - | - | - |
| Day2 | - | - | - | - | 14[12/83] | - | - | 0[0/20] | - | - | - |
| Day3 | - | - | - | - | 6[5/84] | - | - | 0[0/25] | - | - | - |
| **Tanzania** |  |  |  |  |  |  |  |  |  |  |  |
| Day1 | 71[208/294] | 80[4/5] | 48[24/50] | - | 44[81/183] | 62[139/224] | 58[92/160] | - | 30[32/107] | - | - |
| Day2 | 5[15/293] | 0[0/5] | 0[0/50] | - | 0[1/239] | 5[12/256] | 3[4/160] | - | 10[11/106] | - | - |
| Day3 | 1[2/293] | 0[0/5] | 4[2/50] | - | 0[0/249] | 1[2/266] | 0[0/160] | - | 1[1/107] | - | - |
| **Uganda** |  |  |  |  |  |  |  |  |  |  |  |
| Day1 | 6[1/18] | 0[1/288] | 0[0/5] | 85[53/62] | 76[53/70] | 50[51/101] | 76[412/543] | - | - | - | - |
| Day2 | 0[0/18] | 0[0/291] | 0[0/18] | 5[16/342] | 4[15/386] | 3[8/261] | 4[25/635] | - | - | - | - |
| Day3 | 0[0/54] | 0[0/861] | 0[0/40] | 0[1/345] | 0[1/387] | 0[0/261] | 0[0/635] | - | - | - | - |
| **Zambia** |  |  |  |  |  |  |  |  |  |  |  |
| Day1 | - | - | - | 30[8/27] | 49[36/73] | 69[11/16] | 73[46/63] | - | - | - | - |
| Day2 | - | - | - | 0[0/27] | 0[0/73] | 6[1/16] | 5[3/62] | - | - | - | - |
| Day3 | - | - | - | 0[0/27] | 0[0/73] | 0[0/16] | 0[0/62] | - | - | - | - |

**Supplementary Table 2: Early parasitological response (%) for DP by country and year of the study**

| **Country** | **2003** | **2004** | **2005** | **2006** | **2007** | **2008** | **2009** | **2010** | **2011** |
| --- | --- | --- | --- | --- | --- | --- | --- | --- | --- |
| **Burkina Faso** |  |  |  |  |  |  |  |  |  |
| Day1 | - | - | 50[100/199] | - | 40[37/92] | 64[82/129] | - | - | - |
| Day2 | - | - | 0[0/199] | 3[6/184] | 1[1/91] | 1[1/127] | - | - | - |
| Day3 | - | - | 0[0/199] | 0[0/183] | 0[0/92] | 1[1/128] | - | - | - |
| **Cameroon** |  |  |  |  |  |  |  |  |  |
| Day1 | - | - | - | - | 61[34/56] | 71[29/41] | 63[5/8] | - | - |
| Day2 | - | - | - | - | 9[5/56] | 0[0/41] | 0[0/8] | - | - |
| Day3 | - | - | - | - | 0[0/56] | 0[0/41] | 0[0/8] | - | - |
| **Gabon** |  |  |  |  |  |  |  |  |  |
| Day1 | - | - | - | - | 29[2/7] | 42[25/60] | - | - | - |
| Day2 | - | - | - | - | 0[0/6] | 0[0/60] | - | - | - |
| Day3 | - | - | - | - | 0[0/6] | 0[0/60] | - | - | - |
| **Côte d'Ivoire** |  |  |  |  |  |  |  |  |  |
| Day1 | - | - | - | 55[40/73] | 100[1/1] | 57[44/77] | 33[1/3] | - | - |
| Day2 | - | - | - | 6[4/72] | 0[0/1] | 8[6/77] | 0[0/3] | - | - |
| Day3 | - | - | - | 0[0/72] | 0[0/1] | 1[1/76] | 0[0/3] | - | - |
| **Kenya** |  |  |  |  |  |  |  |  |  |
| Day1 | - | - | 67[153/228] | - | 24[17/70] | - | - | - | - |
| Day2 | - | - | 4[10/228] | - | 0[0/70] | - | 7[10/138] | - | - |
| Day3 | - | - | 0[1/227] | - | 0[0/70] | - | 1[1/142] | - | - |
| **Mozambique** |  |  |  |  |  |  |  |  |  |
| Day1 | - | - | 81[56/69] | 94[216/230] | 85[58/68] | 94[126/134] | - | - | - |
| Day2 | - | - | 46[32/69] | 37[85/230] | 21[14/68] | 16[22/134] | - | - | - |
| Day3 | - | - | 9[6/69] | 3[6/230] | 0[0/68] | 0[0/136] | - | - | - |
| **Nigeria** |  |  |  |  |  |  |  |  |  |
| Day1 | - | - | - | - | 95[18/19] | 93[115/124] | - | - | - |
| Day2 | - | - | - | - | 68[13/19] | 29[36/123] | - | - | - |
| Day3 | - | - | - | - | 37[7/19] | 3[4/123] | - | - | - |
| **Rwanda** |  |  |  |  |  |  |  |  |  |
| Day1 | 33[1/3] | - | - | - | 48[13/27] | 28[33/120] | - | - | - |
| Day2 | 7[12/169] | 4[3/82] | - | - | 0[0/27] | 1[1/120] | - | - | - |
| Day3 | 1[1/169] | 0[0/82] | - | - | 0[0/27] | 0[0/120] | - | - | - |
| **Senegal** |  |  |  |  |  |  |  |  |  |
| Day1 | - | - | - | 36[16/45] | 16[3/19] | 18[15/82] | - | - | 52[61/117] |
| Day2 | - | - | - | 0[0/45] | 0[0/19] | 5[4/82] | - | 3[4/120] | 4[5/118] |
| Day3 | - | - | - | 0[0/45] | 0[0/19] | 1[1/80] | - | 0[0/120] | 2[2/119] |
| **Sudan** |  |  |  |  |  |  |  |  |  |
| Day1 | - | - | - | - | - | - | 22[10/46] | - | - |
| Day2 | - | - | - | - | - | - | 0[0/36] | - | - |
| Day3 | - | - | - | - | - | - | 0[0/46] | - | - |
| **Uganda** |  |  |  |  |  |  |  |  |  |
| Day1 | - | - | 73[33/45] | 78[108/138] | 46[77/169] | 66[283/430] | - | - | - |
| Day2 | - | - | 29[13/45] | 5[20/443] | 4[13/325] | 3[16/511] | - | - | - |
| Day3 | - | - | 2[1/45] | 0[1/443] | 0[1/325] | 0[0/511] | - | - | - |
| **Zambia** |  |  |  |  |  |  |  |  |  |
| Day1 | - | - | 15[8/55] | 36[53/147] | 78[14/18] | 51[28/55] | - | - | - |
| Day2 | - | - | 0[0/55] | 1[1/146] | 17[3/18] | 0[0/55] | - | - | - |
| Day3 | - | - | 0[0/55] | 0[0/146] | 0[0/18] | 0[0/55] | - | - | - |

**Supplementary Table 3: Early parasitological response (%) for ASAQ –FDC by country and year of the study**

| **Country** | **2002** | **2003** | **2004** | **2005** | **2006** | **2007** | **2008** | **2009** | **2010** | **2011** |
| --- | --- | --- | --- | --- | --- | --- | --- | --- | --- | --- |
| **Burkina Faso** |  |  |  |  |  |  |  |  |  |  |
| Day1 | - | - | 0[0/1] | - | - | 38[35/92] | 67[136/203] | - | - | 0[0/10] |
| Day2 | - | - | 4[7/159] | 1[1/174] | 2[3/136] | 0[0/92] | 1[3/368] | 0[0/74] | 3[8/230] | 0[0/173] |
| Day3 | - | - | 1[1/158] | 1[1/174] | 0[0/136] | 0[0/92] | 0[1/368] | 0[0/74] | 0[1/230] | 0[0/173] |
| **Cameroon** |  |  |  |  |  |  |  |  |  |  |
| Day1 | - | - | - | - | 73[79/108] | - | - | - | - | - |
| Day2 | - | - | - | - | 8[9/106] | - | - | - | - | - |
| Day3 | - | - | - | - | 0[0/106] | - | - | - | - | - |
| **DRC** |  |  |  |  |  |  |  |  |  |  |
| Day1 | - | - | - | - | - | 8[1/13] | 50[1/2] | - | - | - |
| Day2 | - | - | - | - | - | 0[0/13] | 7[9/122] | 0[0/28] | - | - |
| Day3 | - | - | - | - | - | 0[0/13] | 0[0/123] | 0[0/28] | - | - |
| **Gabon** |  |  |  |  |  |  |  |  |  |  |
| Day1 | - | - | - | - | - | 29[2/7] | 51[35/69] | - | - | - |
| Day2 | - | - | - | - | - | 0[0/8] | 1[1/68] | - | - | - |
| Day3 | - | - | - | - | - | 0[0/9] | 0[0/69] | - | - | - |
| **Côte d'Ivoire** |  |  |  |  |  |  |  |  |  |  |
| Day1 | - | - | - | 1[1/112] | - | - | 7[9/123] | - | - | - |
| Day2 | - | - | - | 0[0/112] | - | - | 0[0/123] | - | - | - |
| Day3 | - | - | - | 0[0/112] | - | - | 0[0/123] | - | - | - |
| **Kenya** |  |  |  |  |  |  |  |  |  |  |
| Day1 | - | - | - | - | - | 100[2/2] | 26[6/23] | - | - | - |
| Day2 | - | - | - | - | - | 0[0/2] | 0[0/23] | - | - | - |
| Day3 | - | - | - | - | - | 0[0/2] | 0[0/23] | - | - | - |
| **Liberia** |  |  |  |  |  |  |  |  |  |  |
| Day1 | - | - | - | - | - | - | - | - | 90[82/91] | 91[10/11] |
| Day2 | - | - | - | - | - | - | 6[14/239] | 1[5/395] | 11[10/91] | 0[0/11] |
| Day3 | - | - | - | - | - | - | 0[0/226] | 0[0/393] | 4[4/91] | 0[0/11] |
| **Madagascar** |  |  |  |  |  |  |  |  |  |  |
| Day1 | - | - | - | - | 37[44/118] | - | - | - | - | - |
| Day2 | - | - | - | - | 3[3/118] | - | - | - | - | - |
| Day3 | - | - | - | - | 0[0/118] | - | - | - | - | - |
| **Mali** |  |  |  |  |  |  |  |  |  |  |
| Day1 | - | - | - | - | 93[124/133] | - | - | - | - | - |
| Day2 | - | - | - | - | 15[20/133] | - | - | - | - | - |
| Day3 | - | - | - | - | 1[1/133] | - | - | - | - | - |
| **Mozambique** |  |  |  |  |  |  |  |  |  |  |
| Day1 | - | - | - | - | - | 89[62/70] | 94[123/131] | - | - | - |
| Day2 | - | - | - | - | - | 20[14/70] | 12[16/131] | - | - | - |
| Day3 | - | - | - | - | - | 0[0/70] | 1[2/135] | - | - | - |
| **Niger** |  |  |  |  |  |  |  |  |  |  |
| Day1 | - | - | - | - | - | - | - | - | - | 74[57/77] |
| Day2 | - | - | - | - | - | - | - | - | - | 11[8/75] |
| Day3 | - | - | - | - | - | - | - | - | - | 4[3/77] |
| **Nigeria** |  |  |  |  |  |  |  |  |  |  |
| Day1 | - | - | - | - | - | 95[18/19] | 92[143/155] | - | - | - |
| Day2 | - | - | - | - | - | 58[11/19] | 27[41/154] | - | - | - |
| Day3 | - | - | - | - | - | 16[3/19] | 4[6/154] | - | - | - |
| **Senegal** |  |  |  |  |  |  |  |  |  |  |
| Day1 | 55[53/96] | 52[133/258] | - | - | 56[145/261] | 66[88/133] | 53[27/51] | - | - | - |
| Day2 | 0[0/96] | 5[14/258] | - | - | 7[18/260] | 4[5/132] | 2[1/51] | - | 1[1/125] | - |
| Day3 | 0[0/96] | 2[4/258] | - | - | 0[1/261] | 0[0/132] | 0[0/51] | - | 0[0/125] | - |
| **Uganda** |  |  |  |  |  |  |  |  |  |  |
| Day1 | - | - | - | - | - | 52[34/66] | 67[196/294] | - | - | - |
| Day2 | - | - | - | - | - | 3[2/65] | 1[4/290] | - | - | - |
| Day3 | - | - | - | - | - | 0[0/66] | 0[1/290] | - | - | - |
| Zambia |  |  |  |  |  |  |  |  |  |  |
| Day1 | - | - | - | - | - | 67[12/18] | 60[38/63] | - | - | - |
| Day2 | - | - | - | - | - | 6[1/18] | 3[2/61] | - | - | - |
| Day3 | - | - | - | - | - | 0[0/18] | 0[0/62] | - | - | - |

**Supplementary Table 4: Early parasitological response (%) for ASAQ–coblistered NFDC by country and year of the study**

| **Country** | **2001** | **2002** | **2003** | **2004** | **2005** | **2006** | **2007** |
| --- | --- | --- | --- | --- | --- | --- | --- |
| **Cameroon** |  |  |  |  |  |  |  |
| Day1 | - | - | - | - | - | - | - |
| Day2 | - | - | - | - | 88[89/101] | - | - |
| Day3 | - | - | - | - | **31[31/101]** | - | - |
| **Ivory Coast** |  |  |  |  |  |  |  |
| Day1 | - | - | - | - | - | 34[27/80] | 0[0/1] |
| Day2 | - | - | - | - | - | 1[1/80] | 0[0/1] |
| Day3 | - | - | - | - | - | 0[0/80] | 0[0/1] |
| **Mali** |  |  |  |  |  |  |  |
| Day1 | - | 89[8/9] | 67[91/135] | 72[68/94] | - | - | - |
| Day2 | - | 0[0/1] | 2[1/45] | 0[0/26] | 4[5/135] | 0[0/101] | - |
| Day3 | - | 0[0/9] | 1[1/135] | 0[0/93] | 1[1/136] | 0[0/101] | - |
| **Senegal** |  |  |  |  |  |  |  |
| Day1 | 72[198/274] | 60[65/109] | 63[60/95] | 65[41/63] | 18[21/118] | 61[40/66] | 31[4/13] |
| Day2 | 4[12/274] | 7[8/109] | 14[13/95] | 19[12/63] | 3[10/331] | 3[2/66] | 0[0/13] |
| Day3 | 0[1/274] | 1[1/109] | 2[2/95] | 0[0/63] | 1[5/442] | 0[0/66] | 0[0/13] |

**Supplementary Table 5: Early Parasitological response (%) for ASAQ –Loose NFDC by country and year of the study**

| **Country** | **1999** | **2000** | **2001** | **2002** | **2003** | **2004** | **2005** | **2006** | **2007** | **2008** | **2012** |
| --- | --- | --- | --- | --- | --- | --- | --- | --- | --- | --- | --- |
| **Angola** |  |  |  |  |  |  |  |  |  |  |  |
| Day1 | - | - | - | - | 0[0/1] | 0[0/1] | - | - | - | - | - |
| Day2 | - | - | - | - | 9[8/93] | 2[1/65] | - | - | - | - | - |
| Day3 | - | - | - | - | 5[5/93] | 2[1/65] | - | - | - | - | - |
| **Burkina Faso** |  |  |  |  |  |  |  |  |  |  |  |
| Day1 | - | - | 15[5/33] | - | - | - | - | - | - | - | - |
| Day2 | - | - | 0[0/33] | - | - | - | - | - | - | - | - |
| Day3 | - | - | 0[0/33] | - | - | - | - | - | - | - | - |
| **Congo** |  |  |  |  |  |  |  |  |  |  |  |
| Day1 | - | - | - | - | - | 100[1/1] | - | - | - | - | - |
| Day2 | - | - | - | - | - | 1[1/100] | - | - | - | - | - |
| Day3 | - | - | - | - | - | 0[0/101] | - | - | - | - | - |
| **DRC** |  |  |  |  |  |  |  |  |  |  |  |
| Day1 | - | - | - | - | - | 67[4/6] | - | - | - | - | - |
| Day2 | - | - | - | - | - | 5[4/88] | - | - | - | - | - |
| Day3 | - | - | - | - | - | 0[0/88] | - | - | - | - | - |
| **Gabon** |  |  |  |  |  |  |  |  |  |  |  |
| Day1 | 49[49/99] | 29[2/7] | - | - | - | - | - | - | - | - | - |
| Day2 | 8[8/95] | 0[0/6] | - | - | - | - | - | - | - | - | - |
| Day3 | 0[0/98] | 0[0/6] | - | - | - | - | - | - | - | - | - |
| **Ghana** |  |  |  |  |  |  |  |  |  |  |  |
| Day1 | - | - | - | - | - | 55[45/82] | - | - | - | - | - |
| Day2 | - | - | - | - | - | 11[9/85] | - | - | - | - | - |
| Day3 | - | - | - | - | - | 2[2/86] | - | - | - | - | - |
| **Guinea** |  |  |  |  |  |  |  |  |  |  |  |
| Day1 | - | - | - | - | - | 14[1/7] | - | - | - | - | - |
| Day2 | - | - | - | - | - | 20[22/110] | - | - | - | - | - |
| Day3 | - | - | - | - | - | 5[5/110] | - | - | - | - | - |
| **Ivory Coast** |  |  |  |  |  |  |  |  |  |  |  |
| Day1 | - | - | - | - | - | - | - | - | - | - | 86[100/116] |
| Day2 | - | - | - | - | - | - | - | - | - | - | 19[21/112] |
| Day3 | - | - | - | - | - | - | - | - | - | - | 1[1/116] |
| **Kenya** |  |  |  |  |  |  |  |  |  |  |  |
| Day1 | 80[86/107] | 73[66/91] | - | - | - | 55[91/164] | 76[28/37] | - | 78[82/105] | 33[8/24] | - |
| Day2 | 12[13/106] | 12[11/91] | - | - | - | 6[10/164] | 11[4/37] | - | 19[20/106] | 0[0/23] | - |
| Day3 | 2[2/105] | 1[1/90] | - | - | - | 2[4/164] | 0[0/37] | - | 3[3/106] | 0[0/25] | - |
| **Liberia** |  |  |  |  |  |  |  |  |  |  |  |
| Day1 | - | - | - | - | - | - | - | - | 4[5/122] | - | - |
| Day2 | - | - | - | - | - | - | - | - | 13[15/119] | - | - |
| Day3 | - | - | - | - | - | - | - | - | 1[1/122] | - | - |
| **Madagascar** |  |  |  |  |  |  |  |  |  |  |  |
| Day1 | - | - | - | - | - | - | - | 63.5[172/271] | 54.2[32/59] | - | - |
| Day2 | - | - | - | - | - | - | - | 13.4[36/269] | 5.1[3/59] | - | - |
| Day3 | - | - | - | - | - | - | - | 3.3[9/269] | 1.7[1/59] | - | - |
| **Mali** |  |  |  |  |  |  |  |  |  |  |  |
| Day1 | - | - | - | - | - | - | - | - | - | - | - |
| Day2 | - | - | - | - | - | - | 11[14/133] | - | - | - | - |
| Day3 | - | - | - | - | - | - | 2[2/133] | - | - | - | - |
| **Nigeria** |  |  |  |  |  |  |  |  |  |  |  |
| Day1 | - | - | - | - | - | - | - | - | 69[20/29] | 71[10/14] | - |
| Day2 | - | - | - | - | - | - | - | - | 15[4/27] | 21[3/14] | - |
| Day3 | - | - | - | - | - | - | - | - | 0[0/31] | 0[0/14] | - |
| **Rwanda** |  |  |  |  |  |  |  |  |  |  |  |
| Day1 | - | - | - | - | - | 0[0/1] | - | - | - | - | - |
| Day2 | - | 100[1/1] | - | - | 12[20/167] | 8[7/83] | - | - | - | - | - |
| Day3 | - | 0[0/1] | - | - | 1[2/167] | 0[0/83] | - | - | - | - | - |
| **Senegal** |  |  |  |  |  |  |  |  |  |  |  |
| Day1 | 81[125/155] | - | - | - | - | - | - | - | - | - | - |
| Day2 | 27[41/154] | - | - | - | - | - | - | - | - | - | - |
| Day3 | 6[9/154] | - | - | - | - | - | - | - | - | - | - |
| **Sierra Leone** |  |  |  |  |  |  |  |  |  |  |  |
| Day1 | - | - | - | - | - | 33[2/6] | - | - | - | - | - |
| Day2 | - | - | - | - | - | 6[7/125] | - | - | - | - | - |
| Day3 | - | - | - | - | - | 6[7/125] | - | - | - | - | - |
| **Sudan** |  |  |  |  |  |  |  |  |  |  |  |
| Day1 | - | - | - | - | 100[1/1] | - | - | - | - | - | - |
| Day2 | - | - | - | - | 18[39/211] | - | - | - | - | - | - |
| Day3 | - | - | - | - | 4[8/211] | - | - | - | - | - | - |
| **Tanzania** |  |  |  |  |  |  |  |  |  |  |  |
| Day1 | - | - | - | 67[132/197] | 56[5/9] | - | - | - | - | - | - |
| Day2 | - | - | - | 9[18/197] | 0[0/9] | - | - | - | - | - | - |
| Day3 | - | - | - | 1[1/197] | 0[0/9] | - | - | - | - | - | - |
| **Uganda** |  |  |  |  |  |  |  |  |  |  |  |
| Day1 | - | - | - | 90[26/29] | 44[45/103] | 100[1/1] | - | 0[0/1] | - | - | - |
| Day2 | - | - | - | 0[0/29] | 4[26/610] | 6[18/308] | 2[6/288] | 4[1/23] | 4[1/26] | 0[0/1] | - |
| Day3 | - | - | - | 3[1/40] | 0[3/630] | 1[2/308] | 0[1/289] | 0[0/23] | 0[0/26] | 0[0/1] | - |

**Supplementary Table 6: Univariable and multivariable risk factors for parasite positivity on day 1**

|  |  |  | Univariable Analysis | | Multivariable Analysis ^c^ | |
| --- | --- | --- | --- | --- | --- | --- |
| Variable | **N (n)** ^a^ | **Random effects ^b^** | **Crude OR [95% CI]** | **P-Value** | **Adjusted OR [95% CI]** | **P-Value** |
| Parasitaemia (2-fold rise) | 16,916(10,099) | 2.860 | 1.52[1.48-1.55] | p<0.001 | 1.49[1.46-1.53] | p<0.001 |
| Baseline Anaemia |  |  |  |  |  |  |
| Non-Anaemic (reference)^d^ | 4,695 (2,748) | 2.409 | 1 | - | - | - |
| Moderate | 6,278 (4,092) |  | 1.18 [1.07-1.3] | 0.001 | 1.21 [1.09-1.34] | p<0.001 |
| Severe | 851 (581) |  | 1.56 [1.30-1.88] | p<0.001 | 1.66 [1.36-2.03] | p<0.001 |
| Unknown | 5,092 (2,678) |  | - | - | - | - |
| Baseline Gametocytaemia |  |  |  |  |  |  |
| No (reference) | 12,251 (7,709) | 2.480 | 1 | - | - |  |
| Yes | 967 (584) |  | 0.91 [0.78-1.06] | 0.238 | - | - |
| Febrile on Presentation (temp > 37.5°c) |  |  |  |  |  |  |
| No (reference) | 5,211 (2,733) | 2.293 | 1 | - | - |  |
| Yes | 11,000 (7,085) |  | 2.22 [2.05-2.42] | p<0.001 | 1.80 [1.65-1.97] | p<0.001 |
| Gender |  |  |  |  |  |  |
| Female (reference) | 7,931 (4,657) | 2.604 | 1 | - | - |  |
| Male | 8,589 (5,184) |  | 1.12 [1.04-1.20] | 0.003 | 1.11 [1.03-1.20] | 0.007 |
| Age Category |  |  |  |  |  |  |
| ≥12 y (reference) | 2,533 (1,171) | 2.644 | 1 | - | - |  |
| <1y | 1,060 (649) |  | 1.96 [1.58-2.43] | p<0.001 | 1.41 [1.11-1.78] | 0.005 |
| 1 to 5 y | 10,053 (6,430) |  | 2.18 [1.87-2.55] | p<0.001 | 1.59 [1.35-1.89] | p<0.001 |
| 5 to 12 y | 3,242 (1,840) |  | 1.47 [1.29-1.68] | p<0.001 | 1.17 [1.01-1.35] | p<0.001 |
| Transmission Settings |  |  |  |  |  |  |
| High (reference) | 5,179 (3,330) | 2.462 | 1 | - | - |  |
| Low/Moderate | 11,737 (6,769) |  | 0.44 [0.23-0.84] | 0.013 | 0.59 [0.30-1.16] | 0.124 |
| Treatment |  |  |  |  |  |  |
| DP (reference) | 3,204 (1,915) | 2.696 | 1 | - | - |  |
| AL | 7,966 (4,721) |  | 1.49 [1.33-1.68] | p<0.001 | 1.57 [1.38-1.78] | p<0.001 |
| ASAQ-FDC | 2,599 (1,620) |  | 1.27 [0.80-2.02] | 0.202 | 1.07 [0.90-1.27] | 0.427 |
| ASAQ-coblisters | 1,057 (623) |  | 0.89 [0.67-1.19] | 0.313 | 1.42 [0.87-2.31] | 0.160 |
| ASAQ-Loose | 2,090 (1,220) |  | 1.11 [0.95-1.30] | 0.429 | 0.75 [0.52-1.08] | 0.125 |

^a^ N=number of patients with non-missing data; n= number of patients with positive blood smear on day 1.

^b^ Estimate of the variance of random effects for the respective univariable analyses

^c^ N=15,793 for the final multivariable model with 9,555 cases of positive parasitaemia. Likelihood ratio test for random effects (*p*<0.001). Variance of random effect = 2.34. Proportion of total variance contributed by the site level variance component (ρ) =0.42. Coefficient (standard error) of intercept = -5.99 (0.3553). The coefficient of variation in parameter estimates was calculated by excluding one study site at a time and expressed as relative standard deviation (%RSD). Distributions of the adjusted odds ratio were generated from 250 bootstrap samples. The %RSD and bootstrap distribution are shown in supplementary file 6 (see table 7 and figure 2).

^d^ Multiple imputation was performed on missing anaemia status using ordinal logistic regression with age, gender and parasitaemia as covariates. The estimates derived using 100 imputations for moderate and severe anaemia are: AOR= 1.13[95% CI: 1.02-1.25], *p*=0.013 and AOR= 1.36[95% CI: 1.14-1.63], *p*=0.001

**Supplementary Table 7: Sensitivity analysis by removing 1 study site at a time for day1 multivariable model**

| Variable | Multivariable Model [95% CI] | Relative Standard Deviation (RSD) ^a^ |
| --- | --- | --- |
| Parasitaemia (10-fold rise) | 3.81 [3.52-4.12] | 0.5% |
| Fever (temp > 37.5 ºC) on presentation | 1.80 [1.65-1.97] | 1.2% |
| Treatment |  |  |
| DP (reference) | 1 | - |
| ASAQ-FDC | 1.07 [0.90-1.27] | 13.6% |
| AL | 1.57 [1.38-1.78] | 2.0% |
| ASAQ-coblistered NFDC | 1.42 [0.87-2.31] | 5.3% |
| ASAQ-loose NFDC | 0.75 [0.52-1.08] | 19.5% |
| Age Category |  |  |
| ≥12 y (reference) | 1 | - |
| <1y | 1.41 [1.11-1.78] | 4.1% |
| 1 to 5 y | 1.59 [1.35-1.89] | 2.5% |
| 5 to 12 y | 1.17 [1.01-1.35] | 5.5% |
| Transmission Settings |  |  |
| High (reference) | 1 | - |
| Low/Moderate | 0.59 [0.30-1.16] | 5.1% |
| Baseline Anaemia |  |  |
| Non-Anaemic (reference) | 1 | - |
| Moderate | 1.21 [1.09-1.34] | 2.9% |
| Severe | 1.66 [1.36-2.03] | 1.6% |
| Gender |  |  |
| Female (reference) | 1 | - |
| Male | 1.11 [1.03-1.20] | 3.80% |

^a^ In order to assess the influence of any given site in parameter estimates, jacknifing was carried out by removing one study site at a time. Relative standard deviation (%RSD) was calculated as

standard deviation divided by the mean estimates of the coefficients obtained by removing each study site at a time.

**Supplementary Table 8: Sensitivity analysis by removing 1 study site at a time for day2 multivariable model**

| Variable | Multivariable Model [95% CI] | Relative Standard Deviation (RSD) ^a^ |
| --- | --- | --- |
| Parasitaemia (10-fold rise) | 2.24 [2.02-2.48] | 0.9% |
| Fever (temp > 37.5 ºC) on presentation | 1.46 [1.28-1.66] | 1.7% |
| Treatment |  |  |
| DP (reference) | 1 | - |
| ASAQ-FDC | 0.90 [0.71-1.14] | 11.3% |
| AL | 1.21 [1.01-1.44] | 5.9% |
| ASAQ-coblistered NFDC | 1.87 [0.86-4.04] | 6.6% |
| ASAQ-loose NFDC | 1.46 [1.05-2.01] | 4.1% |
| Age-category |  |  |
| ≥12 y (reference) | 1 | - |
| <1y | 1.49 [1.09-2.05] | 3.9% |
| 1 to 5 y | 1.54 [1.21-1.97] | 2.7% |
| 5 to 12 y | 1.25 [1.00-1.56] | 4.6% |
| Transmission settings |  |  |
| High (reference) | 1 | - |
| Low/Moderate | 1.88 [1.09-3.24] | 4.1% |
| Baseline anaemia |  |  |
| Non-Anaemic (reference) | 1 | - |
| Moderate | 1.07 [0.94-1.22] | 9.6% |
| Severe | 1.33 [1.06-1.67] | 3.6% |

^a^ In order to assess the influence of any given site in parameter estimates, jacknifing was carried out by removing one study site at a time. Relative standard deviation (%RSD) was calculated as

standard deviation divided by the mean estimates of the coefficients obtained by removing each study site at a time.

**Supplementary Table 9: Sensitivity Analysis by removing 1 study site at a time for day3 multivariable model**

| Variable | Multivariable Model [95% CI] | Relative Standard Deviation (RSD)^a^ |
| --- | --- | --- |
| Parasitaemia (10-fold rise) | 1.65 [1.29-2.13] | 1.6% |
| Fever (temp > 37.5 ºC) on presentation | 1.5 [1.06-2.13] | 3.8% |
| Treatment |  |  |
| DP (reference) | 1 | - |
| ASAQ-FDC | 0.67 [0.36-1.25] | 36.3% |
| AL | 0.93 [0.57-1.52] | 3.6% |
| ASAQ-coblistered NFDC | 2.87 [0.89-9.27] | 3.7% |
| ASAQ-loose NFDC | 2.27 [1.14-4.51] | 5.9% |
| Age category |  |  |
| ≥12 y (reference) | 1 | - |
| <1y | 1.25 [0.62-2.55] | 13.6% |
| 1 to 5 y | 1.09 [0.64-1.87] | 23.5% |
| 5 to 12 y | 1.56 [0.98-2.48] | 3.5% |
| Transmission settings |  |  |
| High (reference) | 1 | - |
| Low/Moderate | 2.71 [1.38-5.36] | 2.4% |
| Baseline anaemia |  |  |
| Non-Anaemic (reference) | 1 | - |
| Moderate | 1.14 [0.8-1.61] | 13.4% |
| Severe | 2.04 [1.21-3.44] | 2.5% |

^a^ In order to assess the influence of any given site in parameter estimates, jacknifing was carried out by removing one study site at a time. Relative standard deviation (%RSD) was calculated as

standard deviation divided by the mean estimates of the coefficients obtained by removing each study site at a time.

**Supplementary Table 10: The effect of weight-adjusted artemisinin dosage on parasite positivity status**

|  | Day1  AOR[95% CI] | Day2  AOR[95% CI] | Day3  AOR[95% CI] |
| --- | --- | --- | --- |
| mg/kg Artemether (/unit increase) | 0.95[0.93-0.99]; *p*=0.003  (N=6299) | 0.99[0.96-1.04]; *p*=0.833  (N=9203) | 0.93[0.82-1.04]; *p*=0.215  (N=9853) |
| mg/kg Dihydroartemisinin (/unit increase) | 0.93[0.88-1.00]; *p*=0.071  (N=2742) | 0.97[0.86-1.08]; *p*=0.604  (N=3689) | 0.81[0.61-1.07]; *p*=0.130  (N=3704) |
| mg/kg Artesunate (/unit increase) | 0.97[0.94-1.02];*p*=0.182  (N=2642) | 0.97[0.92-1.03];*p=*0.351  (N=5865) | 0.93[0.81-1.06]; *p*=0.297  (N=6002) |

The adjusted odds ratio for every unit increase in mg/kg artemesinin derivatives have been derived separately for each of the treatment from a multivariable model adjusting for age, parasitaemia, fever, anaemia, and transmission settings for days 2 and 3. For day1, an additional covariate in gender was added to the model.

**Supplementary Table 11: Multiple imputation of missing covariates**

|  | Final Multivariable Model | Multiple Imputation Model ^b^ | | |
| --- | --- | --- | --- | --- |
| Baseline Anaemia ^a^ | AOR[95% CI] | AOR[95% CI] | Average RVI ^c^ | Largest FMI ^d^ |
| Day1 |  |  |  |  |
| Moderate | 1.21 [1.09-1.34] | 1.13[1.02-1.25] | 0.048 | 0.274 |
| Severe | 1.66 [1.36-2.03] | 1.40[1.16-1.67] |  |  |
|  |  |  |  |  |
| Day2 |  |  |  |  |
| Moderate | 1.07[0.94-1.22] | 1.05[0.93-1.20] | 0.038 | 0.242 |
| Severe | 1.33 [1.06-1.67] | 1.22[0.97-1.55] |  |  |
|  |  |  |  |  |
| Day3 |  |  |  |  |
| Moderate | 1.14 [0.80-1.61] | 1.08 [0.79-1.48] | 0.037 | 0.247 |
| Severe | 2.04 [1.21-3.44] | 1.64[0.99-2.74] |  |  |
| Baseline Fever ^a^ |  |  |  |  |
| Day1 | 1.80 [1.65-1.97] | 1.73[1.58-1.90] | 0.005 | 0.073 |
| Day2 | 1.46 [1.28-1.66] | 1.45[1.28-1.66] | 0.001 | 0.012 |
| Day3 | 1.50 [1.06-2.13] | 1.50[1.06-2.13] | 0.000 | 0.000 |

^a^Age, parasitaemia and gender were used as predictors for baseline anaemia based on ordinal logistic regression. For baseline fever, predictors used were age and baseline parasitaemia based on logistic regression.

*^b^*Multiple Imputation using chained equations were carried out using *mi command* in stata to handle the missing information on anaemia and baseline fever. The adequate number of imputations (*m*) was computed based on largest fraction of missing information (FMI) accepting 5% loss in efficiency using $m\geq100*FMI$. For baseline anaemia and fever, results were summarised from 50 imputations and 20 imputations respectively.

^c^Average Relative Variance Increase (RVI) indicates the increase in variance of the estimates because of the missing values; the closer the values to zero, the smaller the effect of missing values on the variance of the estimates.

^d^The largest fraction of missing information (FMI) is the rate of missing information and reports the largest of all the FMI on coefficient estimates due to missing information.

**Supplementary Table 12: Parameter estimates (standard erros) from overall multivariable model and from model restricted to**

**randomised studies, and studies where quality control was reported on microscopy procedures**

|  | All Data  (N= 27520) | | Randomised studies  (N=22392) | | Studies with QC performed on microscopy (N=17816) | |
| --- | --- | --- | --- | --- | --- | --- |
| Day 3 | $\hat{\beta} \left( se\left( \hat{\beta} \right) \right)$ | *P*-value | $\hat{\beta} \left( se\left( \hat{\beta} \right) \right)$ | *P*-value | $\hat{\beta} \left( se\left( \hat{\beta} \right) \right)$ | *P*-value |
| Intercept | -9.07 | p<0.001 | -8.92 (0.7676) | p<0.001 | -9.17 (0.9041) | p<0.001 |
| Parasitaemia | 0.50 (0.1279) | p<0.001 | 0.48 (0.1382) | p<0.001 | 0.56 (0.1634) | 0.001 |
| Moderate Anaemia | 0.13 (0.1779) | 0.476 | 0.13 (0.1956) | 0.491 | 0.06 (0.2187) | 0.769 |
| Severe Anaemia | 0.71 (0.2667) | 0.008 | 0.79 (0.2864) | 0.006 | 0.87 (0.3324) | 0.009 |
| Fever | 0.41 (0.178) | 0.022 | 0.40 (0.1859) | 0.031 | 0.25 (0.215) | 0.245 |
| <1 y | 0.23 (0.3616) | 0.530 | 0.37 (0.3851) | 0.332 | 0.19 (0.4831) | 0.690 |
| 1 to 5 y | 0.09 (0.2756) | 0.753 | 0.11 (0.2956) | 0.718 | -0.19 (0.409) | 0.635 |
| 5 to 12 y | 0.44 (0.2364) | 0.061 | 0.44 (0.2473) | 0.076 | 0.33 (0.3668) | 0.363 |
| Low/mod transmission | 1.00 (0.3469) | 0.004 | 1.11 (0.3761) | 0.003 | 1.19 (0.4444) | 0.007 |
| AL | -0.07 (0.2491) | 0.774 | -0.09 (0.2532) | 0.737 | -0.3 (0.2987) | 0.315 |
| ASAQ-FDC | -0.40 (0.32) | 0.206 | -0.59 (0.3388) | 0.084 | -0.32 (0.3571) | 0.367 |
| ASAQ-Coblisters | 1.06 (0.598) | 0.078 | 1.17 (0.6296) | 0.062 | 0.29 (0.9238) | 0.757 |
| ASAQ-Loose | 0.82 (0.3515) | 0.020 | 0.61 (0.3704) | 0.099 | 1.01 (0.4775) | 0.035 |
|  |  |  |  |  |  |  |
| Day2 | **All Data**  **(N= 26544)** | | **Randomised studies**  **(N=21405)** | | **Studies with QC performed**  **(N=16863)** | |
|  | $\hat{\beta} \left( se\left( \hat{\beta} \right) \right)$ | *P*-value | $\hat{\beta} \left( se\left( \hat{\beta} \right) \right)$ | *P*-value | $\hat{\beta} \left( se\left( \hat{\beta} \right) \right)$ | *P*-value |
| Intercept | -7.95 |  | -8.16 (0.4068) | p<0.001 | -7.80 (0.4276) | p<0.001 |
| Parasitaemia | 0.81 (0.0532) | p<0.001 | 0.84 (0.0594) | p<0.001 | 0.77 (0.0669) | p<0.001 |
| Moderate Anaemia | 0.07 (0.0667) | 0.2886 | 0.08 (0.0717) | 0.251 | 0.15 (0.0821) | 0.076 |
| Severe Anaemia | 0.28 (0.1162) | 0.01448 | 0.31 (0.1233) | 0.012 | 0.29 (0.1541) | 0.057 |
| Fever | 0.38 (0.0652) | p<0.001 | 0.32 (0.0684) | p<0.001 | 0.38 (0.0814) | p<0.001 |
| <1 y | 0.40 (0.1606) | 0.01251 | 0.43 (0.1783) | 0.015 | 0.18 (0.2146) | 0.414 |
| 1 to 5 y | 0.43 (0.1252) | 0.00052 | 0.52 (0.1422) | 0.000 | 0.22 (0.1778) | 0.225 |
| 5 to 12 y | 0.22 (0.1132) | 0.04846 | 0.31 (0.1297) | 0.016 | 0.13 (0.1575) | 0.405 |
| Low/mod transmission | 0.63 (0.2790) | 0.02406 | 0.66 (0.3262) | 0.043 | 0.8 (0.3273) | 0.015 |
| AL | 0.19 (0.0913) | 0.04022 | 0.20 (0.0958) | 0.035 | 0.16 (0.1061) | 0.120 |
| ASAQ-FDC | -0.10 (0.1191) | 0.38848 | -0.15 (0.1254) | 0.233 | -0.21 (0.1356) | 0.122 |
| ASAQ-Coblisters | 0.62 (0.3937) | 0.11258 | 0.81 (0.4832) | 0.092 | 0.21 (0.5483) | 0.700 |
| ASAQ-Loose | 0.38 (0.1649) | 0.02236 | 0.45 (0.1804) | 0.014 | 0.39 (0.2852) | 0.168 |

**Supplementary Table 13: Combining aggregate data from studies without IPD to the studies with IPD available**

|  | Artemether Lumefantrine | | DHA-Piperaquine | | Artesunate-Amodiaquine (FDC) | |
| --- | --- | --- | --- | --- | --- | --- |
|  | **Meta-Analysis of IPD** | **IPD+ Aggregate Data** | **Meta-Analysis of IPD** | **IPD+ Aggregate Data** | **Meta-Analysis of IPD** | **IPD+ Aggregate Data** |
| Day 1 | 81 sites  I^2^ = 93.7% [92.7; 94.6]  P<0.001  FE= 0.62[0.61; 0.63]  **RE=0.58 [0.52; 0.63]** | 99 sites  I^2^ = 94.3% [93.5; 94.9%]  P<0.001  FE= 0.58 [0.57; 0.59]  **RE=0.56 [0.51; 0.60]** | 25 sites  I^2^ = 95.2% [93.9%; 96.2%]  P<0.001  FE=0.565 [0.545; 0.584]  **RE=0.571 [0.480; 0.658]** | 27 sites  I^2^ = 95.4% [94.2%; 96.3%]  P<0.001  FE=0.572 [0.555; 0.590]  **RE=0.582 [0.497; 0.662]** | 23 sites  I^2^ = 94.4% [92.7%; 95.7%]  P<0.001  FE=0.627 [0.605; 0.649]  **RE=0.649 [0.549; 0.737]** | 29 sites  I^2^ = 95.5% [94.5%; 96.4%]  P<0.001  FE=0.587 [0.567; 0.606]  **RE= 0.605 [0.507; 0.695]** |
| Day 2 | 100 sites  I^2^ = 89.5% [87.8; 91]  P<0.001  FE=0.12 [0.11; 0.13]  **RE=0.04 [0.03; 0.06]** | 123 sites  I^2^ = 90.2% [88.8; 91.4]  P< 0.0001  FE=0.11 [0.11; 0.12]  **RE=0.05 [0.04; 0.06]** | 36 sites  I^2^ = 91.3% [88.9%; 93.1%]  P<0.001  FE=0.147[0.132; 0.163]  **RE=0.043 [0.027; 0.066]** | 39 sites  I^2^ = 91.2% [89%; 93%]  P< 0.0001  FE=0.130 [0.118; 0.142]  **RE=0.046 [0.031; 0.068]** | 32 sites  I^2^ = 88.6% [85%; 91.4%]  P<0.001  FE=0.087 [0.077; 0.099]  **RE=0.046 [0.030; 0.070]** | 39 sites  I^2^ = 92.2% [90.3; 93.8]  P< 0.0001  FE=0.111 [0.100; 0.123]  **RE= 0.048 [0.031; 0.073]** |
| Day 3 | 105 sites  I^2^ = 11.6% [0; 31.4]  P=0.169  FE=0.015 [0.012; 0.018]  **RE=0.013 [0.010; 0.016]** | 133 sites  I^2^ = 67.1% [60.6; 72.5]  P< 0.0001  FE=0.02 [0.018; 0.024]  **RE=0.01 [0.007; 0.012]** | 36 sites  I^2^ = 42.2% [13.9%; 61.2%]  P=0.0047  FE=0.019 [0.014; 0.025]  **RE= 0.009 [0.005; 0.014]** | 42 sites  I^2^ = 67.1% [60.6%; 72.5%]  P=0.0017  FE=0.016 [0.012; 0.021]  **RE=0.008 [0.005; 0.012]** | 32 sites  I^2^ = 37.9% [4.8%; 59.5%]  P=0.017  FE=0.013 [0.009; 0.018]  **RE=0.008 [0.005; 0.013]** | 41 sites  I^2^ = 35.9% [6%; 56.2%]  P=0.0134  FE=0.013 [0.010; 0.018]  **RE=0.008 [0.005; 0.012]** |

**FE**= Fixed Effects; **RE**= Random Effects

**Supplementary Figure 1: Number of patients recruited by year and treatment.**


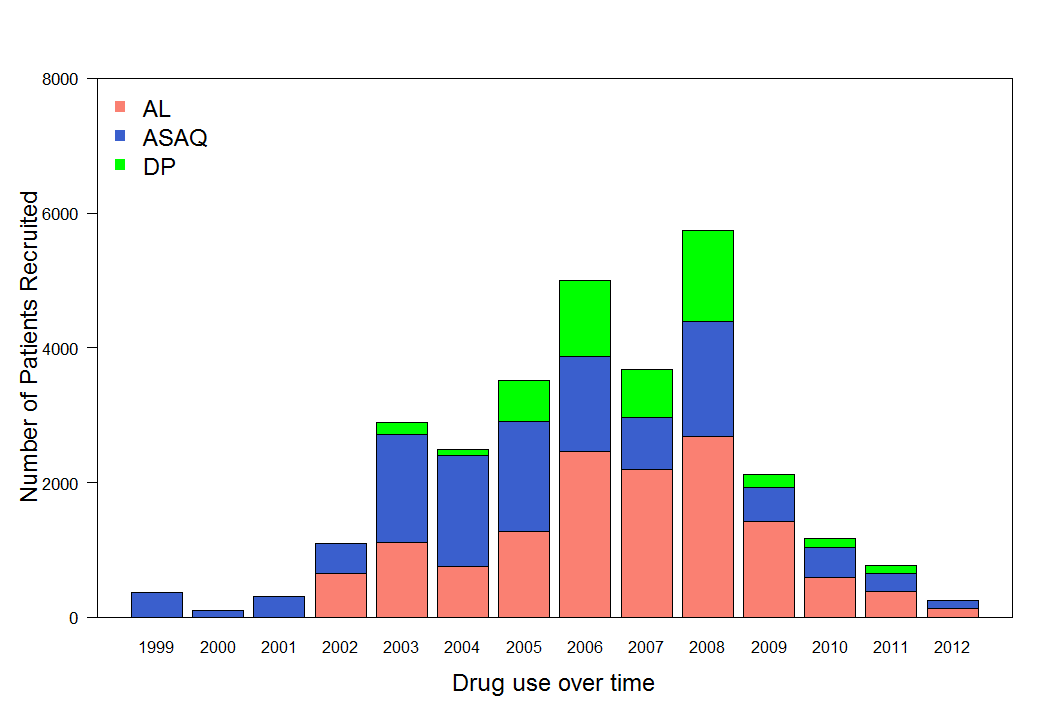


**Supplementary Figure 2: Bootstrap distribution of the parameter estimates for day1 model**


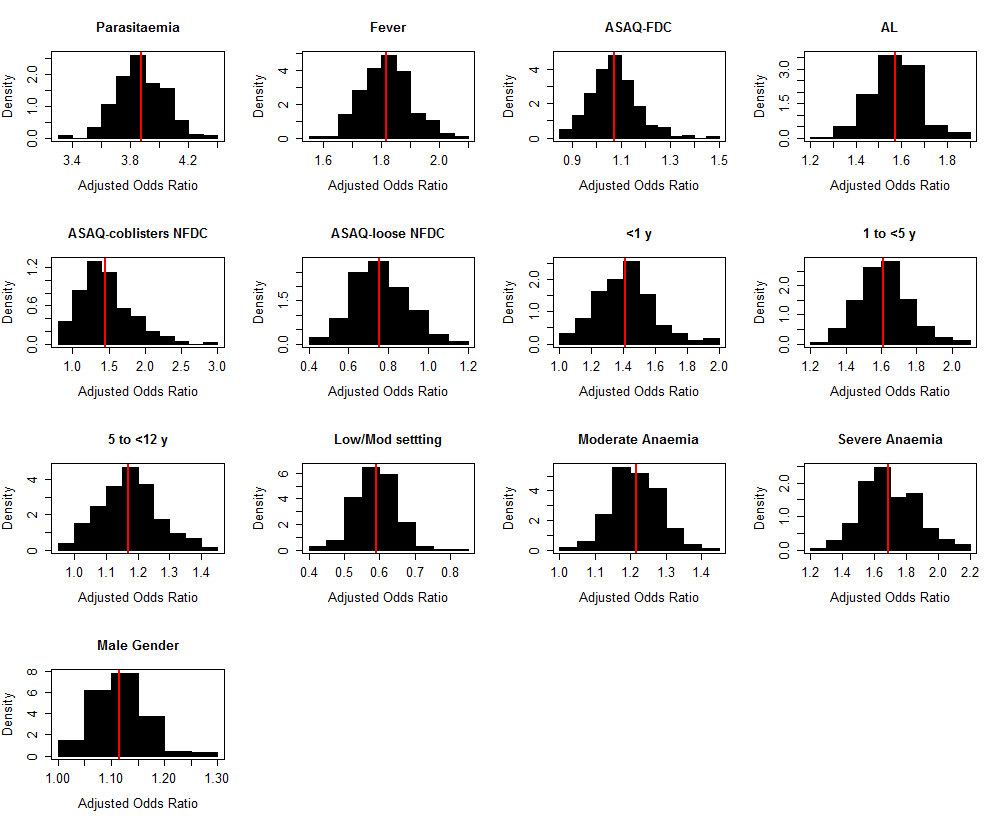


**Legend**: Bootstrapping was performed by taking a random sample of size *n* (n= number of rows in the dataset) from the dataset. This was repeated 250 times. The vertical red line is the mean of the 250 bootstrap samples.

**Supplementary Figure 3: Bootstrap distribution of the parameter estimates for day2 model**


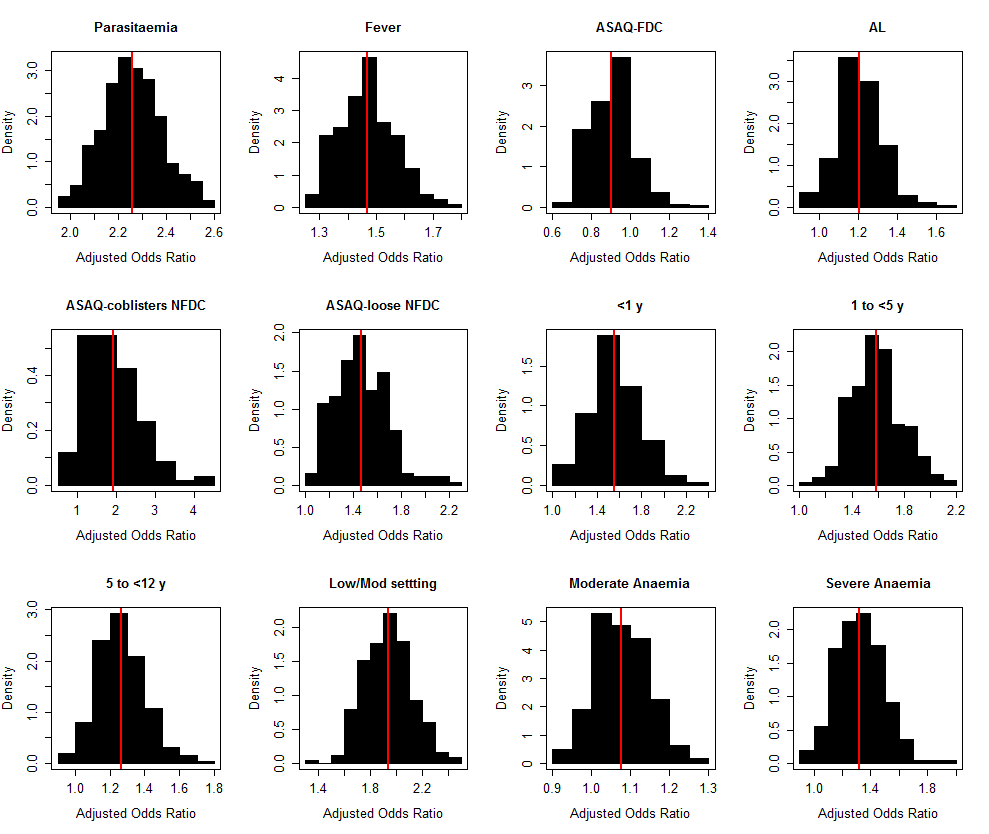


**Legend**: Bootstrapping was performed by taking a random sample of size *n* (n= number of rows in the dataset) from the dataset. This was repeated 250 times. The bold line represents the mean of the 250 bootstrap samples.

Bootstrapping couldn’t be performed for day3 model as the samples had convergence issues resulting in breaking the loops.

**Supplementary Figure 4: Comparing the results generated from IPD analysis to the results reported in publications for day1 PPR**


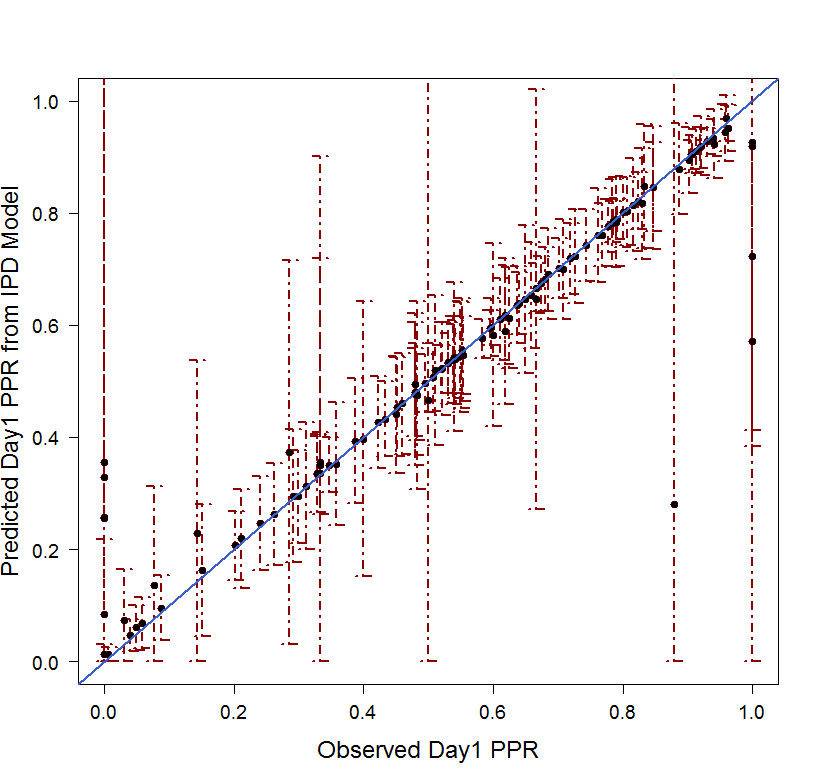


**Legend:** The predicted probability of remaining parasitaemic on day1 was estimated using final fitted random effects multivariable logistic regression: $\hat{p_{i}}=\frac{e^{\hat{\beta}X}}{1+ e^{\hat{\beta}X}}$. Then a binary outcome was simulated from a Bernoulli distribution with this probability, $\hat{p_{i}}$. Overall parasite positivity rate was then estimated for a given study site based on the simulated outcome. This was repeated 1000 times and mean (std dev) PPR was calculated. The y-axis shows the mean PPR from the 1000 simulation runs; the associated error bar shows 95% confidence interval for the simulated mean. The x-axis shows the observed PPR. The diagonal blue line is y=x.

**Supplementary Figure 5: Comparing the results generated from IPD analysis to the results reported in publications for day2 PPR**


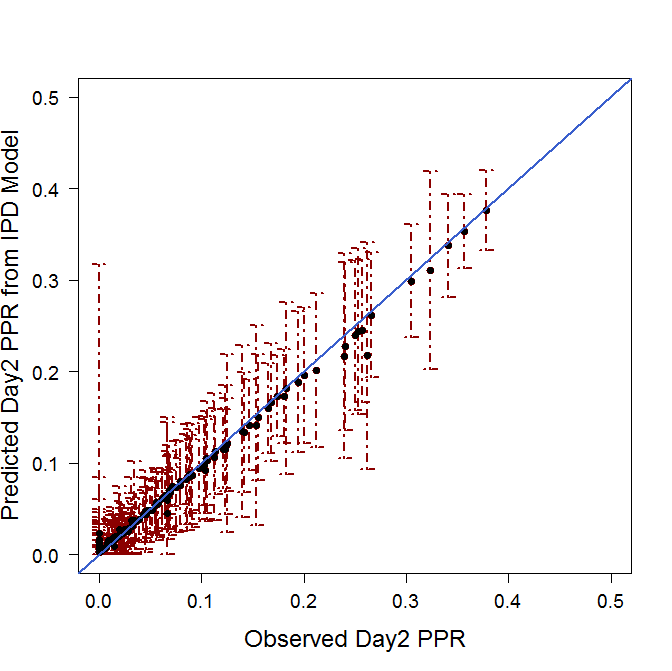


**Legend:** The predicted probability of remaining parasitaemic on day2 was estimated using final fitted random effects multivariable logistic regression: $\hat{p_{i}}=\frac{e^{\hat{\beta}X}}{1+ e^{\hat{\beta}X}}$. Then a binary outcome was simulated from a Bernoulli distribution with this probability, $\hat{p_{i}}$. Overall parasite positivity rate was then estimated for a given study site based on the simulated outcome. This was repeated 1000 times and mean (std dev) PPR was calculated. The y-axis shows the mean PPR from the 1000 simulation runs; the associated error bar shows 95% confidence interval for the simulated mean. The x-axis shows the observed PPR. The diagonal blue line is y=x.

**Supplementary Figure 6: Comparing the results generated from IPD analysis to the results reported in publications for day3 PPR**


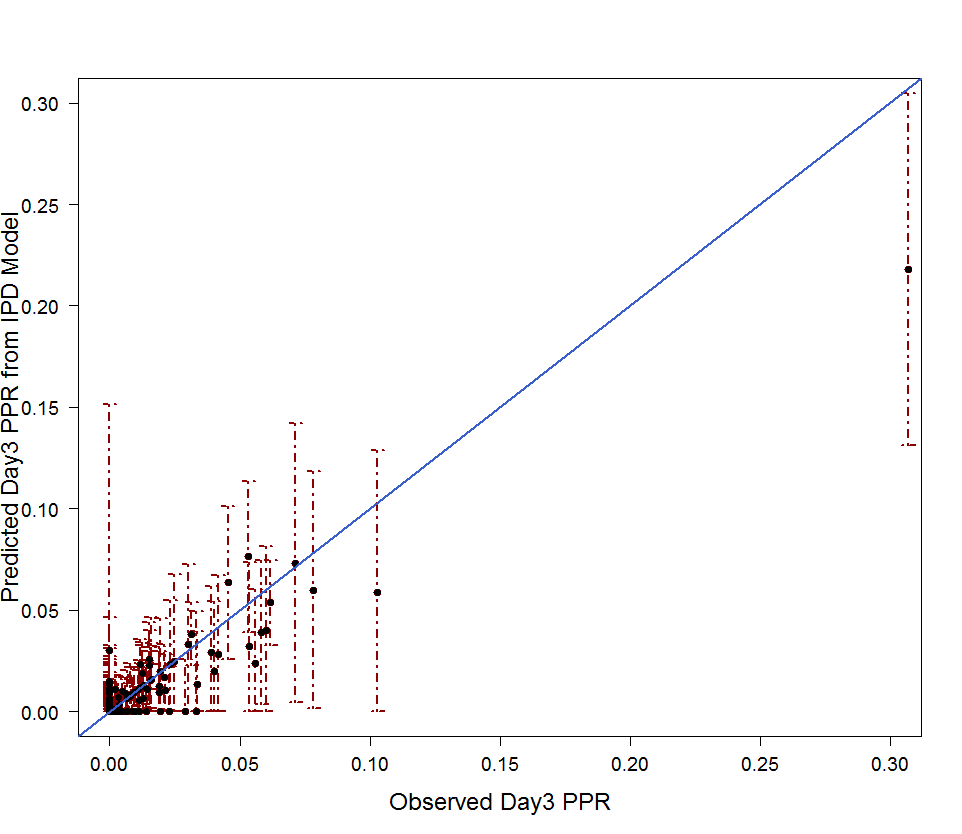


**Legend:** The predicted probability of remaining parasitaemic on day3 was estimated using final fitted random effects multivariable logistic regression: $\hat{p_{i}}=\frac{e^{\hat{\beta}X}}{1+ e^{\hat{\beta}X}}$. Then a binary outcome was simulated from a Bernoulli distribution with this probability, $\hat{p_{i}}$. Overall parasite positivity rate was then estimated for a given study site based on the simulated outcome. This was repeated 1000 times and mean (std dev) PPR was calculated. The y-axis shows the mean PPR from the 1000 simulation runs; the associated error bar shows 95% confidence interval for the simulated mean. The x-axis shows the observed PPR. The diagonal blue line is y=x.

**Supplementary Figure 7: Funnel plot and Egger’s test for assesment of potential publication bias for day1 coefficients**


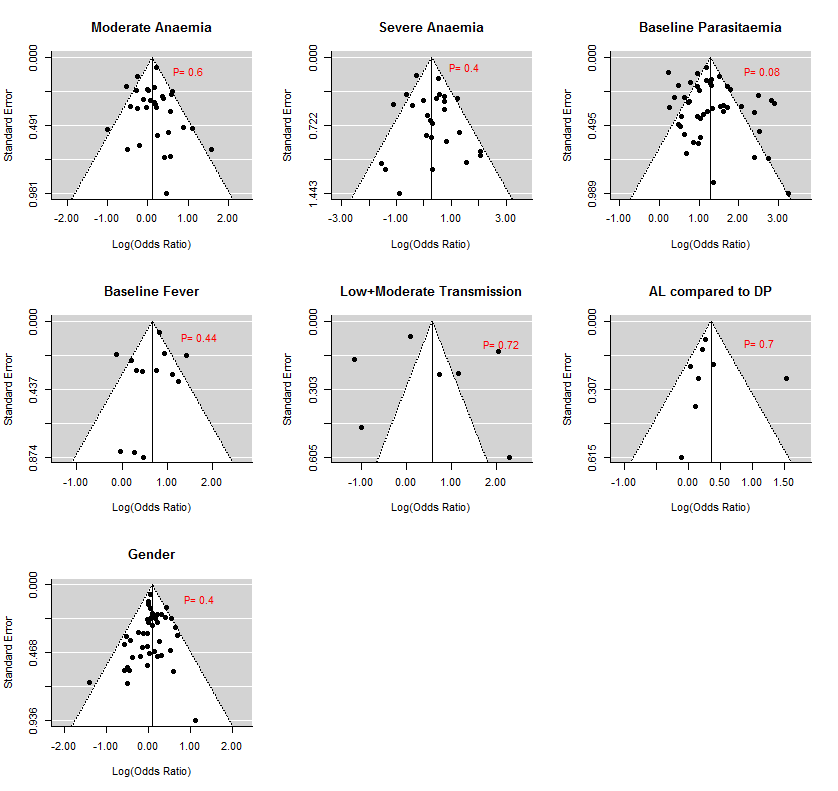


**Legend**: Estimates of the effect size (log-odds ratio and respective standard error) were obtained from each of the studies where there were at least 10 cases of positive parasitaemia for a reliable estimation of coefficients using logistic regression. Meta-analysis was performed using *metafor* package in R. Funnel plot asymmetry was assessed using Egger’s test (shown in red) which suggested no significant evidence of publication bias.

**Supplementary Figure 8: Funnel plot and Egger’s test for assesment of potential publication bias for day2 coefficients**


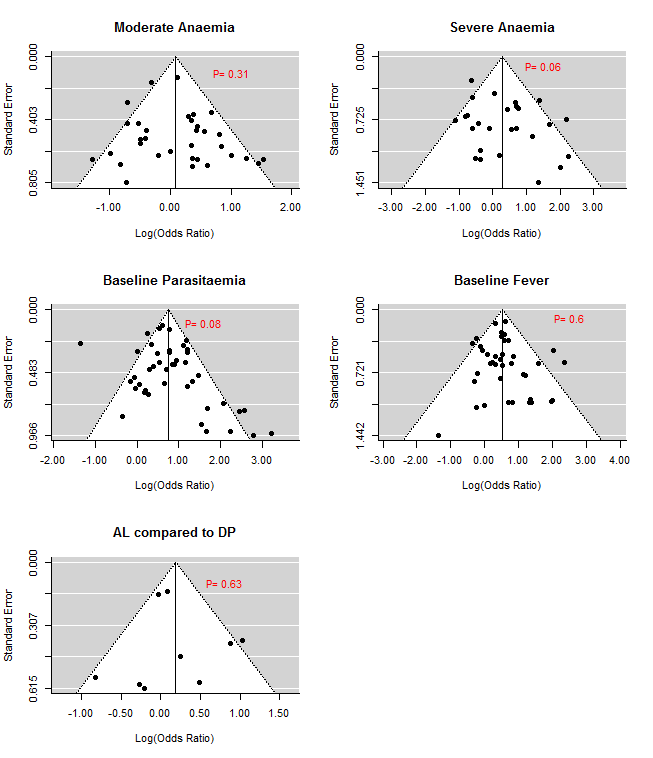


**Legend**: Estimates of the effect size (log-odds ratio and respective standard error) were obtained from each of the studies where there were at least 10 cases of positive parasitaemia for a reliable estimation for coefficients using logistic regression. Meta-analysis was performed using *metafor* package in R. Funnel plot asymmetry was assessed using Egger’s test (shown in red) which suggested no significant evidence of publication bias. Such analysis couldn’t be performed for examining the effect sizes for day3 model because of the low number of events (253 cases of positive parasitaemia on day3).
